# FMRP regulates an ethanol-dependent shift in GABA_B_R function and expression with rapid antidepressant properties

**DOI:** 10.1038/ncomms12867

**Published:** 2016-09-26

**Authors:** Sarah A. Wolfe, Emily R. Workman, Chelcie F. Heaney, Farr Niere, Sanjeev Namjoshi, Luisa P. Cacheaux, Sean P. Farris, Michael R. Drew, Boris V. Zemelman, R. Adron Harris, Kimberly F. Raab-Graham

**Affiliations:** 1 Waggoner Center for Alcohol and Addiction Research, The University of Texas at Austin, Austin, Texas 78712, USA; 2 Center for Learning and Memory, The University of Texas at Austin, Austin, Texas 78712, USA; 3 Institute of Cell and Molecular Biology, The University of Texas at Austin, Austin, Texas 78712, USA; 4 Department of Neuroscience, The University of Texas at Austin, Austin, Texas 78712, USA; 5 Department of Physiology and Pharmacology, Wake Forest University School of Medicine, Winston-Salem, North Carolina 27101, USA

## Abstract

Alcohol promotes lasting neuroadaptive changes that may provide relief from depressive symptoms, often referred to as the self-medication hypothesis. However, the molecular/synaptic pathways that are shared by alcohol and antidepressants are unknown. In the current study, acute exposure to ethanol produced lasting antidepressant and anxiolytic behaviours. To understand the functional basis of these behaviours, we examined a molecular pathway that is activated by rapid antidepressants. Ethanol, like rapid antidepressants, alters γ-aminobutyric acid type B receptor (GABA_B_R) expression and signalling, to increase dendritic calcium. Furthermore, new GABA_B_Rs are synthesized in response to ethanol treatment, requiring fragile-X mental retardation protein (FMRP). Ethanol-dependent changes in GABA_B_R expression, dendritic signalling, and antidepressant efficacy are absent in *Fmr1-*knockout (KO) mice. These findings indicate that FMRP is an important regulator of protein synthesis following alcohol exposure, providing a molecular basis for the antidepressant efficacy of acute ethanol exposure.

## Abstract (web summary)

Alcohol is thought to lead to neuroadaptive changes, although the underlying molecular mechanisms are unclear. Here, the authors find ethanol treatment alters GABA_B_-receptor expression via fragile-X mental retardation protein in mice, leading to antidepressant-like behaviours.

## Graphical abstract

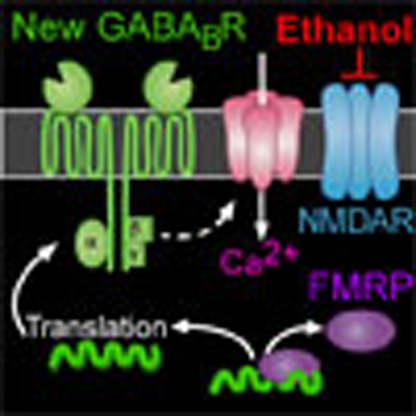

The presence of major depression increases the risk of alcohol use disorders (AUDs) by ∼2-fold (and vice versa)^1^. The self-medication hypothesis suggests AUDs may develop when the initial antidepressant actions of alcohol are shifted to depressant allostatic states with chronic abuse^2^. The molecular mechanism underlying the initial antidepressant effects of alcohol is unknown.

A major advance in understanding and treating depression is the recognition that NMDA receptor (NMDAR) antagonists act as rapid and effective antidepressant drugs^3^. A single injection of an NMDAR antagonist or ‘rapid antidepressant' is effective within 2 h and has sustained antidepressant efficacy for 2 weeks^4^. These long-lasting properties depend on the activity of mammalian target of rapamycin (mTOR)^5^,^6^, a serine/threonine kinase essential for messenger RNA translation^7^. Recently, we demonstrated that activation of mTOR-dependent protein synthesis by NMDAR antagonists requires a shift in GABA_B_R signalling from opening potassium channels to facilitating an increase in dendritic calcium^6^,^8^. Interestingly, both acute ethanol and rapid antidepressants block NMDARs^4^,^9^. In light of these data, we propose that ethanol has lasting antidepressant efficacy, shares the same downstream molecular signalling events as rapid antidepressants, and requires *de novo* protein synthesis (Supplementary Fig. 1).

Studies suggest that antidepressant efficacy requires two phases—an induction phase and a sustained phase^10^,^11^. Notably, GABA_B_R-mediated, mTORC1-dependent protein synthesis is required for the long-lasting sustained phase of rapid antidepressants. Our previous work indicates that both new protein synthesis and an increase in protein stability are required for the GABA_B_R shift in function necessary to increase mTORC1 activity^8^. However, the mechanism that initiates such dynamic changes in protein expression by rapid antidepressants remains unclear.

FMRP is an RNA-binding protein that has been characterized as a repressor of mRNA translation. Some forms of synaptic activity trigger FMRP to release its targets, allowing them to be translated^12^,^13^. Moreover, degradation and new protein synthesis of FMRP creates a window for the translation of specific mRNAs, facilitating long-lasting changes in synaptic function^14^,^15^. Complete loss of FMRP results in Fragile-X syndrome (FXS), the single most common genetic cause of autism^16^. Moreover, reduced levels of FMRP, caused by a pre-mutation, lead to a higher incidence of tremors, ataxia, memory loss, and neuronal neuropathy in older men^17^. These findings argue that precise levels of FMRP protein and its target mRNAs are required for normal neuronal function.

Drugs of abuse promote profound changes in gene expression, mRNA translation rates and synaptic protein composition^18^,^19^. Some studies suggest that drugs and alcohol highjack the molecular mechanisms that underlie synaptic plasticity^20^,^21^. In agreement with this premise, FMRP has been implicated in cocaine addiction^22^. However, little is known about the mRNA targets and the signalling mechanisms involved. Here we describe a critical role for FMRP in mediating GABA_B_R synthesis and plasticity following acute ethanol exposure, a mechanism required for antidepressant efficacy.

## Results

### Antidepressant and anxiolytic effects of ethanol on behaviour

**Figure 1:**
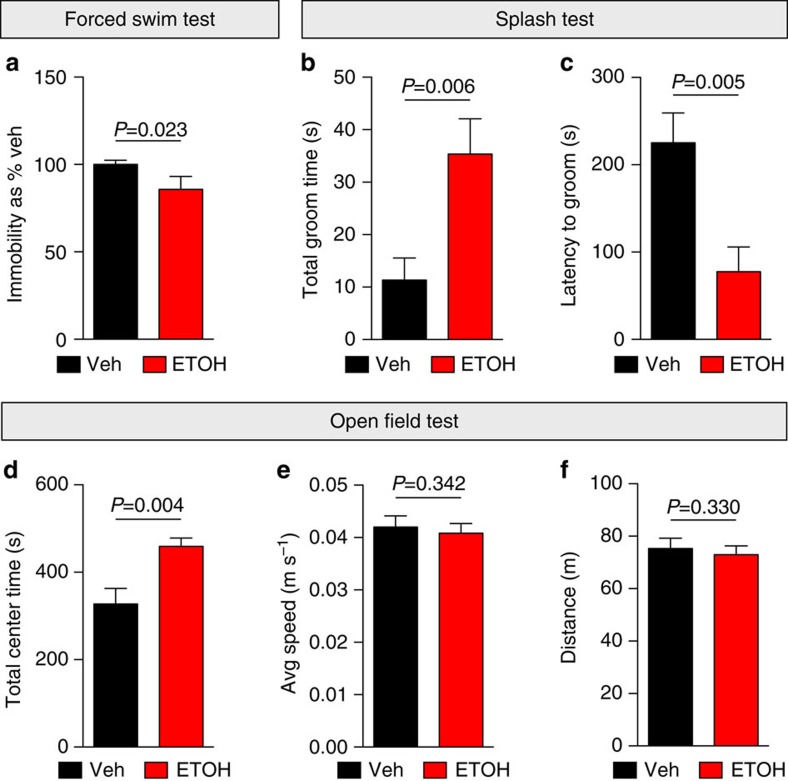
Ethanol elicits a lasting antidepressant-like effect on behaviour. (**a**) C57BL/6 male mice were subjected to the forced swim test 24 h after i.p. injection with vehicle (Veh; saline) or ethanol (ETOH; 2.5 g kg^−1^). Ethanol treatment reduced immobility, indicating antidepressant efficacy. Veh=100±2.5, *n*=10 mice; ETOH=86±7.4, *n*=6. (**b**,**c**) In the splash test, male C57BL/6 mice groomed longer and took less time to initiate grooming 24 h post ethanol (2.5 g kg^−1^, i.p.) compared with 24 h post-vehicle (saline, i.p.) treatment. Total groom time: Veh=11.34±4.23 s, *n*=6; ETOH=35.37±6.72 s, *n*=5. Latency to groom: Veh=225.2±34.13 s, *n*=6; ETOH=77.55±28.44 s, *n*=5. (**d**–**f**) Total centre time, speed and distance were measured in the open field test 24 h post injection. Ethanol-treated (2.5 g kg^−1^, i.p.) mice spent more time in the centre, while speed and distance were unaffected compared with vehicle-treated (saline, i.p.) mice, indicating an ethanol-induced anxiolytic effect without altering mobility. Total centre time: Veh=327.5±35.62 s, *n*=6; ETOH=459.2±19.13 s, *n*=6. Average speed: Veh=0.042±0.002 m s^−1^, *n*=6; ETOH=0.041±0.002 m s^−1^, *n*=6. Total distance: Veh=75.35±3.92 m, *n*=6; ETOH=73.00±3.35 m, *n*=6. Significance determined by one-tailed *t*-test. Values represent mean±s.e.m.

To determine if acute alcohol has antidepressant properties, as predicted by the self-medication hypothesis, we first assessed the efficacy of alcohol on antidepressant- and anxiolytic-like effects on behaviour. The forced swim test (FST) is a rodent behavioural test predictive of antidepressant activity in humans^23^. Rodents treated with a single injection of NMDAR antagonists or rapid antidepressants swim longer and thus have reduced immobility relative to controls. Notably, these positive effects on behaviour last long after the drug has been metabolized^5^,^6^,^8^,^24^. Therefore, we considered the possibility that ethanol, which blocks NMDARs^9^, could also act like an antidepressant at 24 h, well beyond the intoxication period^25^. To test this, C57BL/6 mice were injected with ethanol (2.5 g kg^−1^, intraperitoneal (i.p.)), a concentration that is achieved during self-administration in mice^26^. Twenty-four hours after injection, the immobility of ethanol-treated mice was reduced by ∼15% relative to controls (Fig. 1a), similar to our previous observation in mice that had been exposed to the rapid antidepressant Ro-25-6981 (refs 6, 8). These results demonstrate that acute ethanol elicits a lasting antidepressant effect on behaviour similar to that seen with rapid antidepressants^8^.

As another measure of antidepressant effect of ethanol on behaviour, we assessed the grooming behaviour of mice using the splash test after ethanol or saline administration. The splash test measures latency to groom and dedicated grooming time as indicators of self-care and motivational behaviour^27^,^28^. Lack of self-care is often observed in humans with depressive disorder^29^. We have previously shown that mice receiving a single i.p. injection of the rapid antidepressant Ro-25-6981 spend more time grooming compared with control mice^8^. We hypothesized that ethanol would produce similar effects on grooming behaviour. Indeed, ethanol-treated mice spent more time grooming and displayed shorter latency to initiate grooming relative to controls (Fig. 1b,c).

Ethanol is a well-known anxiolytic substance^30^. However, the anxiolytic effect of a single dose of ethanol 24 h after administration has not been determined. We subjected ethanol- and saline-injected mice to the open field test to assess the influence of ethanol on anxiety-like behaviours after 24 h. Mice that spend more time in the centre of the open field are scored as having reduced anxiety-like behaviour relative to mice that remain close to the perimeter^31^. Indeed, mice that received a single dose of ethanol (2.5 g kg^−1^, i.p.) had reduced anxiety-like behaviour, spending ∼40% more time in the centre relative to controls (Fig. 1d). There was no significant difference in total distance travelled or average speed between the groups (Fig. 1e,f). These data suggest that the anxiolytic effects of ethanol last up to 24 h post injection.

### Acute ethanol increases GABA_B_R2 and surface GABA_B_Rs

Both ethanol and rapid antidepressants block NMDARs in the hippocampus^4^,^9^. One of the first events triggered by NMDAR antagonism is increased dendritic GABA_B_R2 protein expression^8^. GABA_B_Rs are obligate heteromultimers, consisting of GABA_B_R1 and R2. GABA_B_R2 is required for expression of receptors at the surface by masking an endoplasmic reticulum retention sequence on GABA_B_R1 (ref. 32). Similarly, treatment with a rapid antidepressant leads to (1) increased dendritic expression of GABA_B_R2 but not GABA_B_R1 (ref. 8) and (2) a corresponding increase in surface expression of GABA_B_R1 (refs 6, 8).

**Figure 2:**
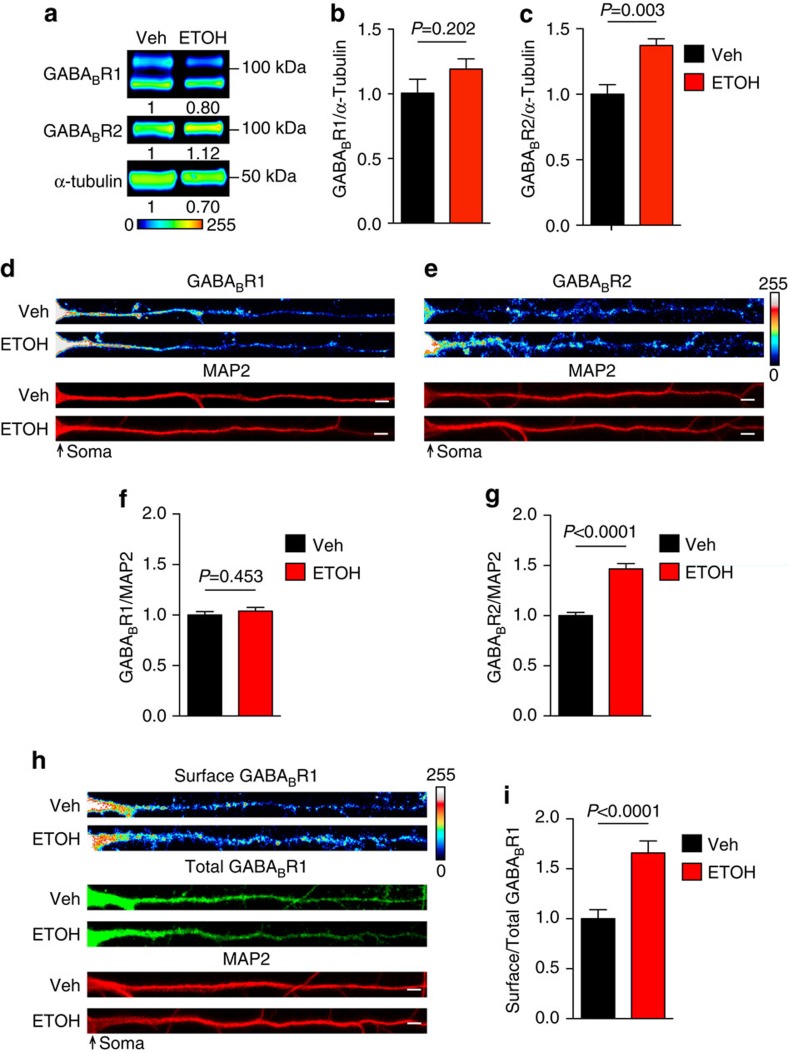
Acute ethanol increases dendritic GABA_B_Rs in hippocampus. (**a**–**c**) Western blot analyses of GABA_B_R1 and GABA_B_R2 in isolated hippocampal synaptoneurosomes from ethanol-treated (ETOH; 2.5 g kg^−1^ i.p.), and vehicle-treated (Veh; saline i.p.) C57BL/6 male mice 30 min post injection. (**a**) Pseudocoloured representative western blots to show intensity with normalized optical density for each band indicated below blot (Lookup table, below western blot). No significant change was observed in **b** GABA_B_R1, but a significant increase was found in **c** GABA_B_R2 with ethanol treatment. Western blots were normalized to the loading control, α-Tubulin. GABA_B_R1: Veh=1.00±0.11; ETOH=1.19±0.08. Experiment was repeated five times. GABA_B_R2: Veh=1.00±0.07; ETOH=1.37±0.05. (**d**,**e**) Representative immunostaining images of GABA_B_R1 and GABA_B_R2 in cultured rat hippocampal dendrites normalized to microtubule associated protein 2 (MAP2) as volume control. There was no change in **f** GABA_B_R1 and a significant increase in **g** GABA_B_R2 in ethanol treated (30 mM, 2 h) compared with vehicle-treated (H2O, 2 h) dendrites: Total GABA_B_R1: Veh=1.00±0.03, *n*=46 dendrites; ETOH=1.04±0.04, *n*=51 dendrites. Total GABA_B_R2: Veh= 1.00±0.03, *n*=46 dendrites; ETOH=1.47±0.05, *n*=51 dendrites. (**h**,**i**) Immunofluorescence shows a significant increase in surface GABA_B_R1 expression in dendrites of cultured rat hippocampal neurons treated with ethanol (30 mM, 2 h); (**i**) Surface expression of GABA_B_R1 in vehicle-treated (H_2_O, 2 h) and ethanol-treated (30 mM, 2 h) dendrites. Veh=1.00±0.09, *n*=43 dendrites; ETOH=1.66±0.12, *n*=47 dendrites. Significance determined by Student's *t*-test. Values represent mean±s.e.m. Scale bars, 5 μm. Uncropped version of western blots, with size markers are available in Supplementary Fig. 7a.

To determine if acute ethanol exposure *in vivo* rapidly increases the levels of GABA_B_R1 and/or GABA_B_R2, hippocampal synaptoneurosomes were isolated from mice that had been injected with a single dose of ethanol (2.5 g kg^−1^, i.p.) or saline for western blot analysis. The hippocampi were collected within the initiation phase (30 min post injection), a phase where molecular changes facilitate increased downstream mTORC1 activity^11^. Consistent with rapid antidepressants, acute ethanol injection increased the protein expression of GABA_B_R2 by ∼37% in the hippocampus with no significant change in GABA_B_R1 (Fig. 2a–c; uncropped blots, Supplementary Fig. 7a).

To further identify the subcellular localization of ethanol-induced increase in GABA_B_R2, we examined GABA_B_R expression in cultured hippocampal neurons. GABA_B_R1 and R2 were immunostained and quantified in the dendrites. A concentration of 30 mM ethanol was chosen, as it has been shown to reduce NMDAR activity in hippocampal neurons and reflects that achieved *in vivo* following i.p. injection^9^,^33^. Acute ethanol exposure (30 mM, 2 h) increased the dendritic levels of GABA_B_R2 by ∼47%, but did not affect GABA_B_R1 levels (Fig. 2d–g). We did not observe a difference in the diameter of the primary dendrites between vehicle- and ethanol-treated neurons, demonstrating that ethanol does not modify dendritic calibre (Supplementary Fig. 2). These *in vivo* and *in vitro* findings establish a role for ethanol in increasing GABA_B_R2 protein expression.

Since GABA_B_R2 is required for the surface expression of the heteromultimeric receptor, we predicted that the ethanol-induced elevation in GABA_B_R2 levels would increase expression of receptors at the surface. We measured the surface expression of dendritic GABA_B_Rs using an antibody directed against the extracellular domain of GABA_B_R1 in unpermeabilized hippocampal neurons. The surface signal was normalized by the total dendritic GABA_B_R1 levels after permeabilization^6^. As predicted, surface expression of GABA_B_Rs in ethanol-treated neurons was significantly higher (∼66% increase) relative to controls (Fig. 2h,i). This ethanol effect was consistent with what we previously observed following rapid antidepressant treatment of cultured hippocampal neurons^6^. Collectively, these results suggest that ethanol promotes the surface expression of GABA_B_Rs, and this is likely achieved by increasing GABA_B_R2 protein levels.

### FMRP regulates GABA_B_R1 and GABA_B_R2 expression

Next, we sought to identify the mechanism by which NMDAR antagonism increases GABA_B_R2 expression. GABA_B_R2 mRNA is present in the dendrites of hippocampal neurons^34^, suggesting that this mRNA may be locally regulated at the translational level. Thus, we examined RNA-binding factors that may regulate GABA_B_R2 mRNA expression in dendrites. Notably, both GABA_B_R1 and GABA_B_R2 mRNAs are reported targets of FMRP, an RNA-binding protein that stalls translational elongation of its targets^35^,^36^.

**Figure 3:**
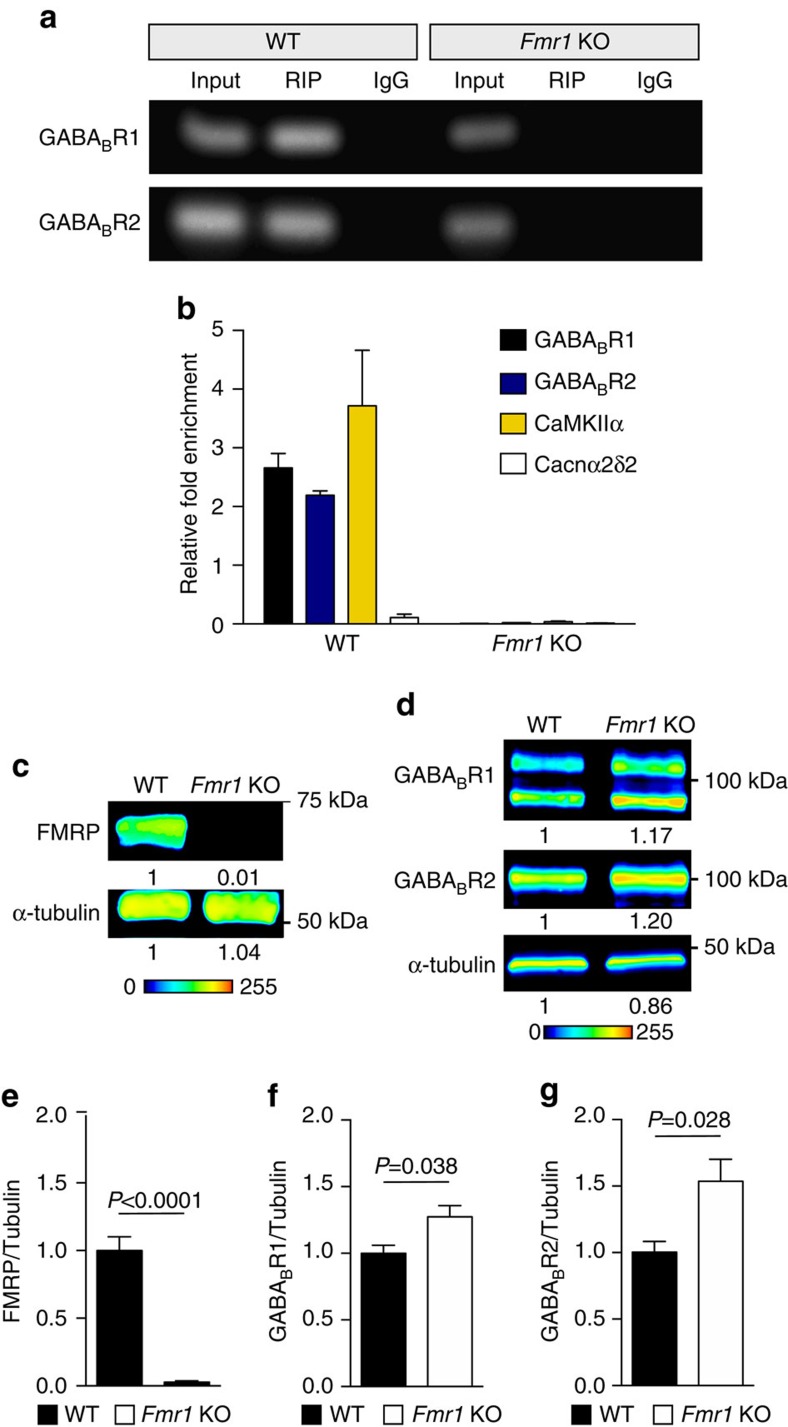
GABA_B_R1 and GABA_B_R2 mRNAs are FMRP targets. (**a**,**b**) RNA immunoprecipitation (RIP) for FMRP was performed using brains from wild type (WT) and *Fmr1* KO male mice. (**a**) Gels showing RT–qPCR amplified product of input sample, FMRP RIP, and IgG control for GABA_B_R1 and GABA_B_R2. (**b**) Relative fold-enrichment as determined by real-time qPCR relative to input control (ΔΔCt=2^−(Ct FMRP RIP−Ct IgG RIP)−(Ct FMRP input−Ct IgG input)^). FMRP binds GABA_B_R1, GABA_B_R2, and the positive control CaMKIIα mRNA as detected in the RIP sample by real-time qPCR. Cacnα2δ2 served as a negative control and was not detected above background. WT: GABA_B_R1=2.66±0.248, *n*=2; GABA_B_R2=2.19±0.08, *n*=2; CaMKII=3.72±0.94, *n*=2; Cacnα2δ2=0.11±0.6, *n*=2. *Fmr1* KO: GABA_B_R1=0.01±0.0002, *n*=2; GABA_B_R2=0.02±0.00006, *n*=2; CaMKII±0.04±0.01, *n*=2; Cacnα2δ2=0.012±0.005, *n*=2. (**c**–**g**) Western blot analysis of hippocampal synaptoneurosomes isolated from C57BL/6 WT and *Fmr1* KO mice on a C57BL/6 background indicates the absence of (**e**) FMRP and increased protein expression of (**f**) GABA_B_R1 and (**g**) GABA_B_R2. Representative western blots are pseudocoloured to indicate intensity of bands, and the normalized optical density for each band is indicated below blot (Lookup table, below western blot). Western blots were normalized to the loading control, α-Tubulin. WT: FMRP=1.00±0.10; GABA_B_R1=1.00±0.06; GABA_B_R2=1.00±0.08. *Fmr1* KO: FMRP=0.03±0.01; GABA_B_R1=1.27±0.08; GABA_B_R2=1.54±0.17. Experiment was repeated three times. Significance determined by Student's *t*-test. Values represent mean±s.e.m. Uncropped versions of qPCR gel, with size markers, are available in Supplementary Fig. 7d. Uncropped version of western blots, with size markers are available in Supplementary Fig. 7b.

To test the hypothesis that FMRP regulates GABA_B_R mRNA translation, we first verified that (1) GABA_B_R mRNAs bind to FMRP, and that (2) the absence of FMRP in knockout mice results in aberrant expression of GABA_B_Rs. Using a specific antibody against FMRP, bound mRNAs were isolated using RNA immunoprecipitation (RIP). GABA_B_R1 and GABA_B_R2 binding were assessed by reverse transcription (RT) and quantitative PCR. Indeed, GABA_B_R1 and GABA_B_R2 mRNAs were detected in the immunoprecipitate, along with CaMKIIα, a well-known FMRP mRNA target (Fig. 3a,b; uncropped representative qPCR gels, Supplementary Fig. 7d). The calcium channel accessory subunit Cacnα2δ2 mRNA is not a reported target for FMRP^35^ and was used as a negative control. Cacnα2δ2 mRNA was not detected in the FMRP RIP (Fig. 3b). In parallel, we used lysates isolated from brains of mice with a genetic deletion of the *Fmr1* gene. We did not observe amplification of any of the mRNAs in *Fmr1* KO brains, providing additional evidence for specific binding of FMRP to GABA_B_R1 and GABA_B_R2 mRNAs (Fig. 3a–c; uncropped blots, Supplementary Fig. 7b).

Next, we determined if FMRP regulates GABA_B_R1 and GABA_B_R2 protein levels. Genetic deletion of *Fmr1* leads to the constitutive translation of FMRP target mRNAs and the loss of activity-dependent translation^16^. Protein levels of GABA_B_R1 and GABA_B_R2 were compared in hippocampal synaptoneurosomes from *Fmr1* KO and wild-type (WT) mice (Fig. 3c–g). GABA_B_R2 basal protein levels were elevated by ∼53% in *Fmr1* KO hippocampi (Fig. 3g). GABA_B_R1 protein levels also increased, albeit to a lesser extent than GABA_B_R2 (Fig. 3f). Collectively, these data suggest that FMRP regulates the expression of GABA_B_R1 and GABA_B_R2.

### Ethanol and rapid antidepressants reduce dendritic FMRP

**Figure 4:**
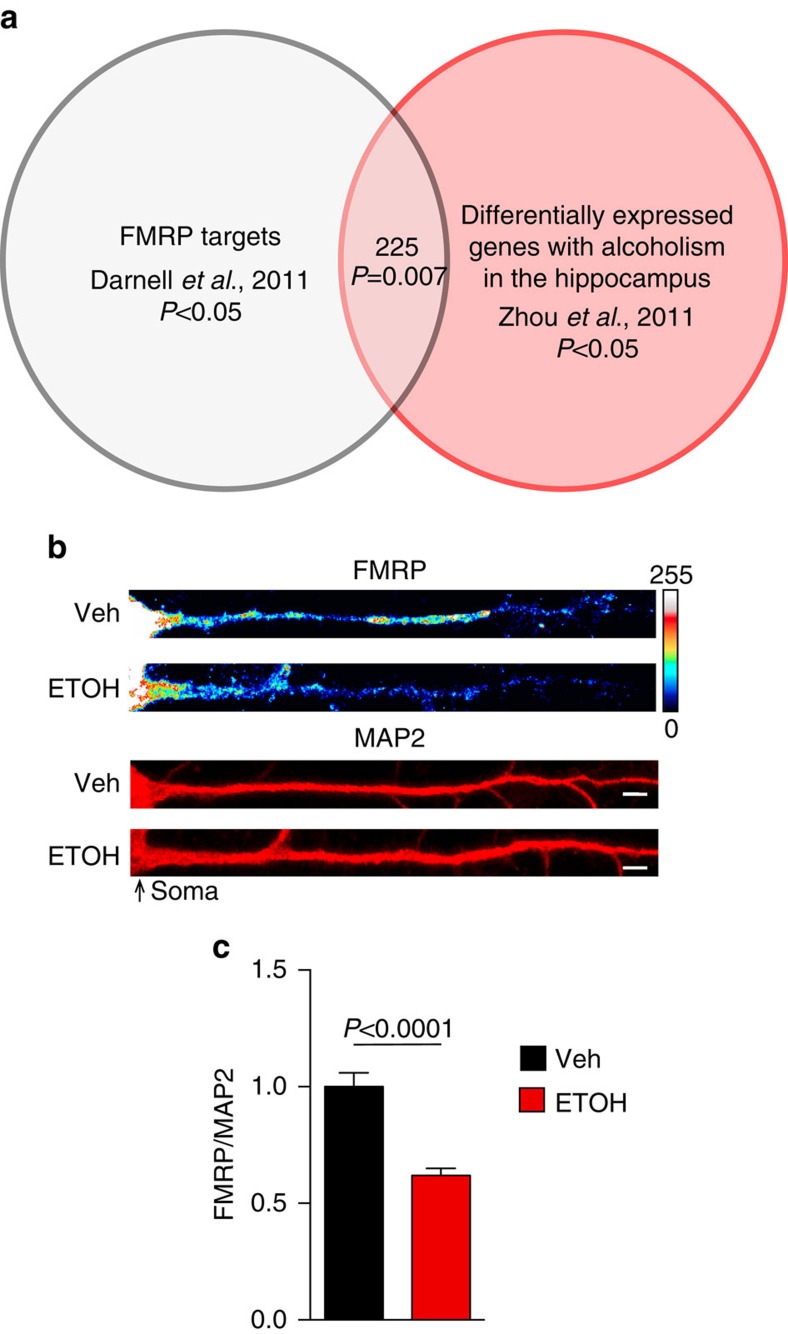
FMRP and AUD share target mRNAs and ethanol decreases FMRP. (**a**) Venn diagram illustrating the significant overlap between FMRP mRNA targets and differentially expressed genes in the hippocampus of humans with AUDs. Significance determined with *χ*^2^-test. (**b**,**c**) Immunofluorescence images normalized to MAP2 as volume control and quantification summary shows a significant decrease in FMRP expression in dendrites of cultured hippocampal neurons treated with ethanol (ETOH; 30 mM, 2 h) compared with vehicle treated (Veh; H_2_O, 2 h). Veh: FMRP =1.00±0.06, *n*=32 dendrites; ETOH: FMRP=0.62±0.03, *n*=31 dendrites. Significance determined by Student's *t*-test. Values represent mean±s.e.m. Scale bar, 5 μm.

As an initial test to determine if FMRP-regulated translation is linked to alcohol exposure, we compared FMRP target mRNAs^35^ with mRNAs that are differentially expressed in the hippocampi of alcohol-dependent humans^37^. Remarkably, 225 or ∼25% of verified FMRP target mRNAs overlap with mRNAs that are altered in alcohol-dependent individuals, suggesting a role for FMRP in aberrant protein levels observed in humans with AUD (Fig. 4a)^35^,^37^. We then determined whether exposure to acute ethanol (30 mM, 2 h) or Ro-25-6981 (10 μM, 2 h) alters FMRP expression in the dendrites of hippocampal neurons. Using immunofluorescence, we found that ethanol and Ro-25-6981 reduced FMRP levels by ∼38% and 45%, respectively (ethanol: Fig. 4b,c; Ro-25-6981: Supplementary Fig. 3). These data suggest that ethanol and Ro-25-6981 alter protein expression in an FMRP-dependent manner.

### Ethanol-induced synthesis of GABA_B_R2 requires FMRP

**Figure 5:**
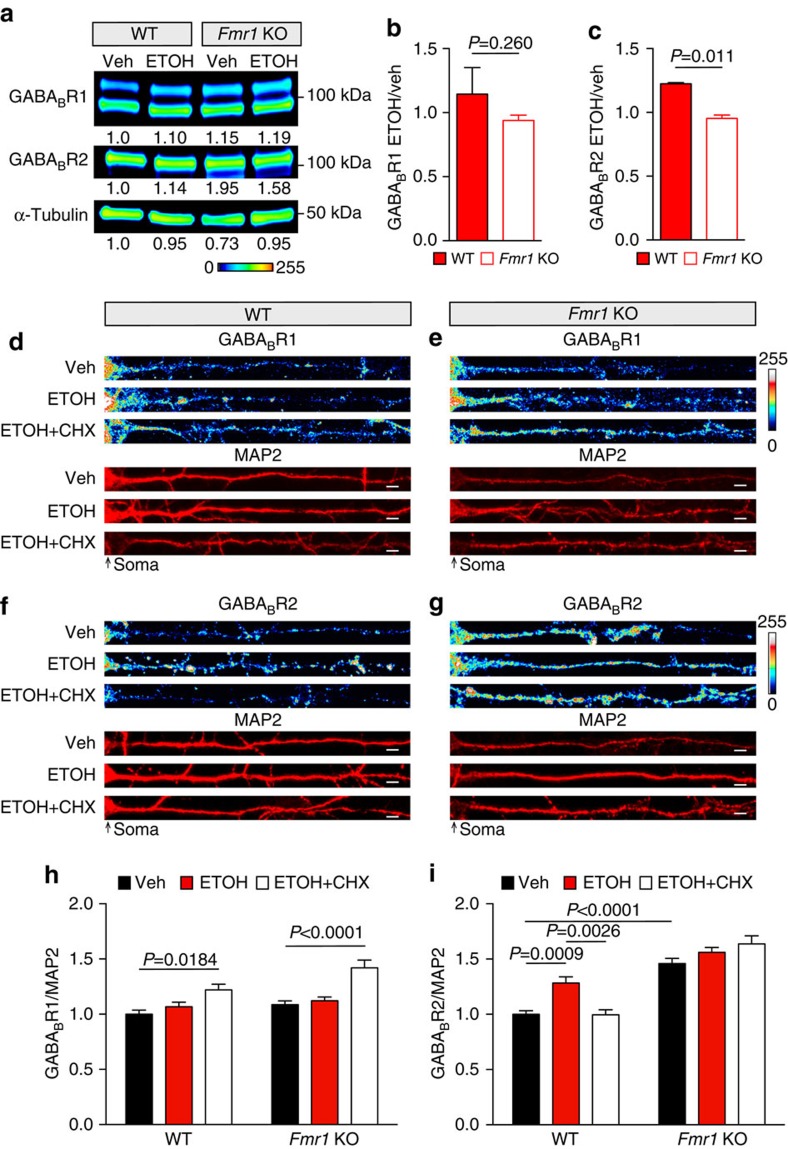
*Fmr1* KO prevents ethanol-induced altered GABA_B_R expression. (**a**–**c**) Western blot analysis of GABA_B_R1 and GABA_B_R2 in wild type (WT) and *Fmr1* KO C57BL/6 hippocampal synaptoneurosomes after vehicle (Veh; saline i.p., 30 min) or ethanol (ETOH; 2.5 g kg^−1^ i.p., 30 min) treatment. (**a**) Pseudocoloured representative western blots showing band intensity, and normalized optical densities to WT–vehicle are reported below each image (lookup table, below western blot). Western blots were normalized to the loading control, α-Tubulin. No change was found in **b** GABA_B_R1 after ethanol treatment in either genotype as shown by ethanol/vehicle comparison. A significant increase in **c** GABA_B_R2 expression was observed in WT mice after ethanol, but no change was observed in *Fmr1* KO mice (shown as ethanol/vehicle). WT ETOH/Veh: GABA_B_R1=1.15±0.21; GABA_B_R2=1.22±0.01. *Fmr1* KO ETOH/Veh: GABA_B_R1=0.94±0.04; GABA_B_R2=0.95±0.03. Experiment was repeated three times. Significance determined by Student's *t*-test. Values represent mean±s.e.m. Representative immunofluorescent images (**d**–**g**) and quantification summaries (**h**,**i**) of dendritic expression of GABA_B_R1 and GABA_B_R2 from WT and *Fmr1* KO primary mouse hippocampal cultures normalized to MAP2. (**h**) GABA_B_R1 expression was not changed in either genotype after 2-h treatment with vehicle (Veh; H_2_O), ethanol (ETOH; 30 mM), or ethanol and cycloheximide (30 mM ETOH+50 μM CHX). WT GABA_B_R1: Veh=1.00±0.04, *n*=44 dendrites; ETOH=1.07±0.04, *n*=29 dendrites; ETOH+CHX=1.22±0.05, *n*=34 dendrites. *Fmr1* KO GABA_B_R1: Veh=1.09±0.03, *n*=72 dendrites; ETOH=1.12±0.03, *n*=41 dendrites; ETOH+CHX=1.42±0.07, *n*=43 dendrites. (**i**) GABA_B_R2 expression in WT neurons increased after ethanol (ETOH; 30 mM, 2 h) compared with vehicle (Veh; H_2_O, 2 h) treatment, and was rescued with co-treatment of cycloheximide (CHX; 50 μM, 2 h). GABA_B_R2 expression in *Fmr1* KO dendrites was not significantly altered between neurons treated with Veh, ETOH, or ETOH+CHX. WT GABA_B_R2: Veh=1.00±0.03, *n*=41 dendrites; ETOH=1.28±0.06, *n*=40 dendrites; ETOH+CHX=0.99±0.05, *n*=33 dendrites. *Fmr1* KO GABA_B_R2: Veh=1.46±0.05, *n*=73 dendrites; ETOH=1.56±0.04, *n*=45 dendrites; ETOH+CHX=1.63±0.07, *n*=36 dendrites. Significance determined by two-way analysis of variance with Tukey's post test. Value represent mean±s.e.m. Scale bars, 5 μm. Uncropped version of western blots, with size markers are available in Supplementary Fig. 7c.

Due to the ethanol-induced decreases in FMRP, we hypothesized that FMRP is required for ethanol-induced expression of GABA_B_Rs. Specifically, if expression of GABA_B_Rs is constitutive and unregulated in *Fmr1* KO mice, then ethanol-induced changes in GABA_B_R expression should be absent in *Fmr1* KO mice. Hippocampal synaptoneurosomes were isolated from WT and *Fmr1* KO mice 30 min after i.p. injection of ethanol (2.5 g kg^−1^). Western blot analysis indicated that both GABA_B_R1 and GABA_B_R2 expression remained constant in vehicle- and ethanol-treated *Fmr1* KO mice. As observed in Fig. 2, WT hippocampal synaptoneurosomes showed an ∼23% increase in GABA_B_R2 but no change in GABA_B_R1 expression (Fig. 5a–c; uncropped blots, Supplementary Fig. 7c). These data suggest that ethanol-induced changes in GABA_B_R expression are dependent on FMRP translational regulation.

To determine whether protein synthesis is essential for the FMRP-dependent changes in GABA_B_R expression, we measured ethanol-induced GABA_B_Rs in the presence of cycloheximide (CHX), a protein synthesis inhibitor. As demonstrated previously, ethanol did not influence the dendritic expression of GABA_B_R1; however, co-treatment with cycloheximide increased GABA_B_R1 expression by ∼22%. FMRP deletion did not affect the basal, ethanol-, or cycloheximide-induced dendritic protein expression of GABA_B_R1 (Fig. 5d,e,h). For GABA_B_R2, we again saw a significant ∼28% increase in dendritic expression with acute ethanol treatment; however, in the presence of cycloheximide the ethanol-induced increase was abolished. Notably in *Fmr1* KO cultures, no change was observed with ethanol or ethanol+cycloheximide (Fig. 5f,g,i).

**Figure 6:**
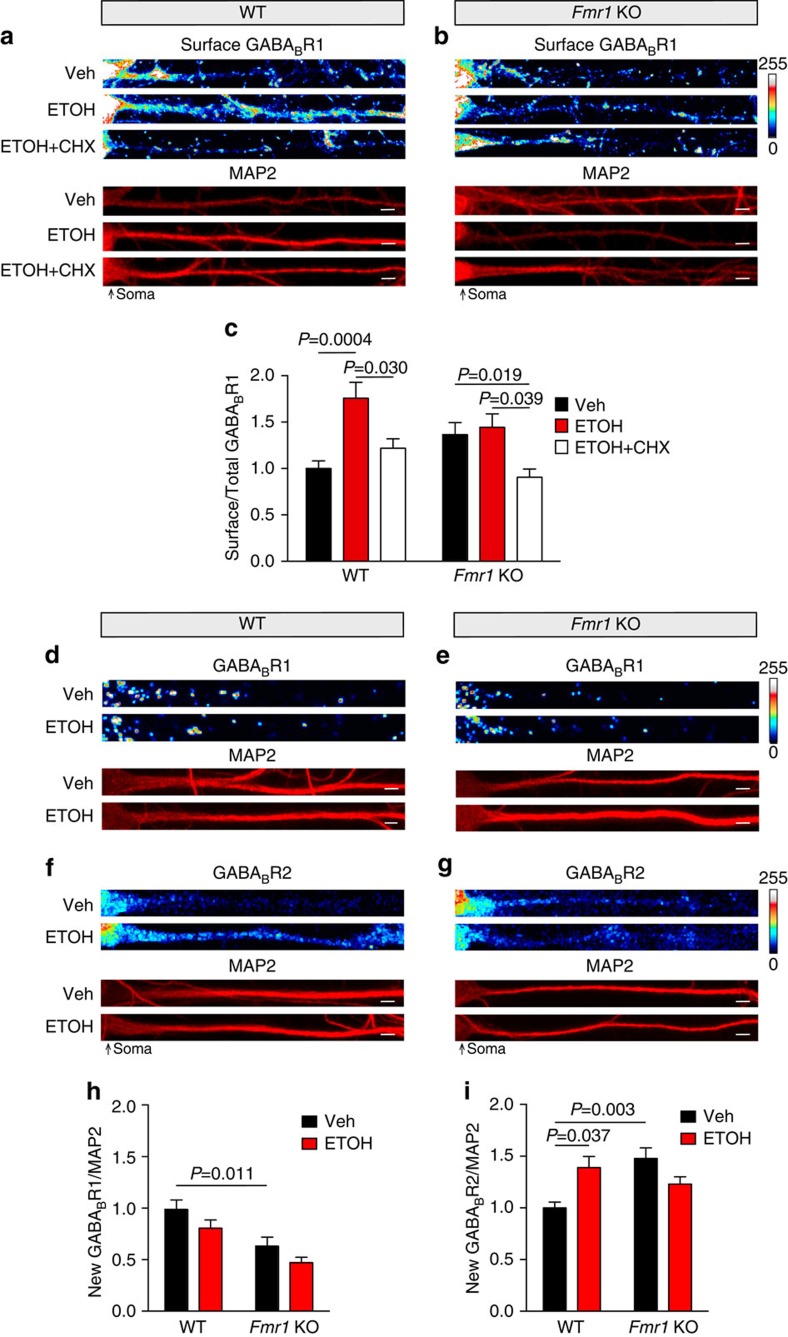
New GABA_B_R2 protein and surface expression requires FMRP. Immunofluorescent images and quantification summary of GABA_B_R1 surface expression in wild type (WT) and *Fmr1* KO primary hippocampal cultures normalized to MAP2 as volume control. (**a**,**b**) Representative images of immunostaining. (**c**) Increased expression of surface GABA_B_R1 in WT dendrites after ethanol (ETOH: 30 mM, 2 h) compared with vehicle (Veh: H_2_O, 2 h) or ethanol–cycloheximide (30 mM ETOH+50 μM CHX, 2 h) treatment. No significant change in surface GABA_B_R1 expression in *Fmr1* KO cultures treated with ETOH or ETOH+CHX was observed. WT surface GABA_B_R1: Veh=1.00±0.08, *n*=28 dendrites; ETOH=1.76±0.17, *n*=37 dendrites; ETOH+CHX=1.22±0.1, *n*=36 dendrites. *Fmr1* KO surface GABA_B_R1: Veh=1.36±0.13, *n*=39 dendrites; ETOH=1.44±0.15, *n*=42 dendrites; ETOH+CHX=0.91±0.09, *n*=29 dendrites. (**d**–**i**) BONCAT combined with PLA, a method to detect newly synthesized proteins. (**d**–**g**) Representative images for GABA_B_R1 and GABA_B_R2 expression. Pixels were equally dilated by 1 using ImageJ software for enhanced visualization as described by Cajigas *et al*.^34^. In WT and *Fmr1* KO primary hippocampal cultures (**h**) GABA_B_R1 synthesis in dendrites was not altered by ethanol (30 mM, 2 h) compared with vehicle (H_2_O, 2 h) treatment normalized to MAP2. WT GABA_B_R1: Veh=1.00±0.09, *n*=47 dendrites; ETOH=0.82±0.08, *n*=39 dendrites. *Fmr1* KO GABA_B_R1: Veh=0.64±0.09, *n*=38 dendrites; ETOH=0.48±0.05, *n*=36 dendrites. (**i**) In contrast, ethanol induced a significant increase in new GABA_B_R2 synthesis in WT hippocampal dendrites but not in *Fmr1* KO dendrites. WT GABA_B_R2: Veh=1.00±0.06, *n*=21 dendrites; ETOH=1.39±0.11, *n*=25 dendrites. *Fmr1* KO GABA_B_R2: Veh=1.48±0.10, *n*=32 dendrites; ETOH=1.23±0.07, *n*=41 dendrites. Significance determined by two-way analysis of variance with Tukey's post test. Values represent mean±s.e.m. Scale bars, 5 μm.

We next examined the requirement for protein synthesis and FMRP in ethanol-dependent surface expression of GABA_B_Rs. Using WT and *Fmr1* KO hippocampal neurons, we measured ethanol-induced surface expression of GABA_B_R1 with or without cycloheximide. Co-assembly of GABA_B_R1 and R2 is required to express GABA_B_R heterodimers in the membrane^32^. Thus, we predicted that the ethanol-induced increase in surface GABA_B_Rs would require FMRP-regulated synthesis of GABA_B_R2. Again, acute ethanol increased dendritic surface GABA_B_Rs by ∼76%, and this was blocked by cycloheximide. In *Fmr1* KO neurons there was no significant ethanol-induced change in surface GABA_B_Rs, but a decrease was observed with ethanol+cycloheximide (Fig. 6a–c). These data suggest that GABA_B_R2 protein synthesis is required for the increased surface expression of the hetermultimeric receptor with ethanol exposure.

FMRP is reported to repress new protein synthesis. Considering the effects of the protein synthesis inhibitor, these data suggest that the ethanol-mediated reduction in FMRP results in the increase in dendritic GABA_B_R2 by *de novo* protein synthesis. To provide more direct evidence, we performed bioorthogonal noncanonical amino-acid tagging (BONCAT) in conjunction with proximity ligation assay (PLA-Duolink)^38^. BONCAT+PLA can be used to detect new synthesis of proteins of interest, such as GABA_B_R1 and GABA_B_R2. Through click chemistry, noncanonical amino acids that are incorporated during mRNA translation are biotinylated. PLA, on the other hand, generates a fluorescent signal when two antibodies are within 30–40 nm of each other (that is, anti-GABA_B_R1 or GABA_B_R2 and anti-biotin). By combining these methods, we determined that ethanol treatment increases new protein synthesis of GABA_B_R2 by ∼40%, but does not alter GABA_B_R1 synthesis, similar to the GABA_B_R changes induced by rapid antidepressants^8^. In *Fmr1* KO dendrites, basal levels of GABA_B_R2s increased by ∼48%, while a significant decrease was observed in GABA_B_R1 levels. In addition, ethanol-induced translation of GABA_B_R2 was lost in *Fmr1* KO dendrites (Fig. 6d–i). These data provide additional evidence that the ethanol-induced increase in GABA_B_R2 expression is due to new protein synthesis that requires the release of translational repression by FMRP.

### Ethanol-induced GABA_B_R plasticity requires FMRP

**Figure 7:**
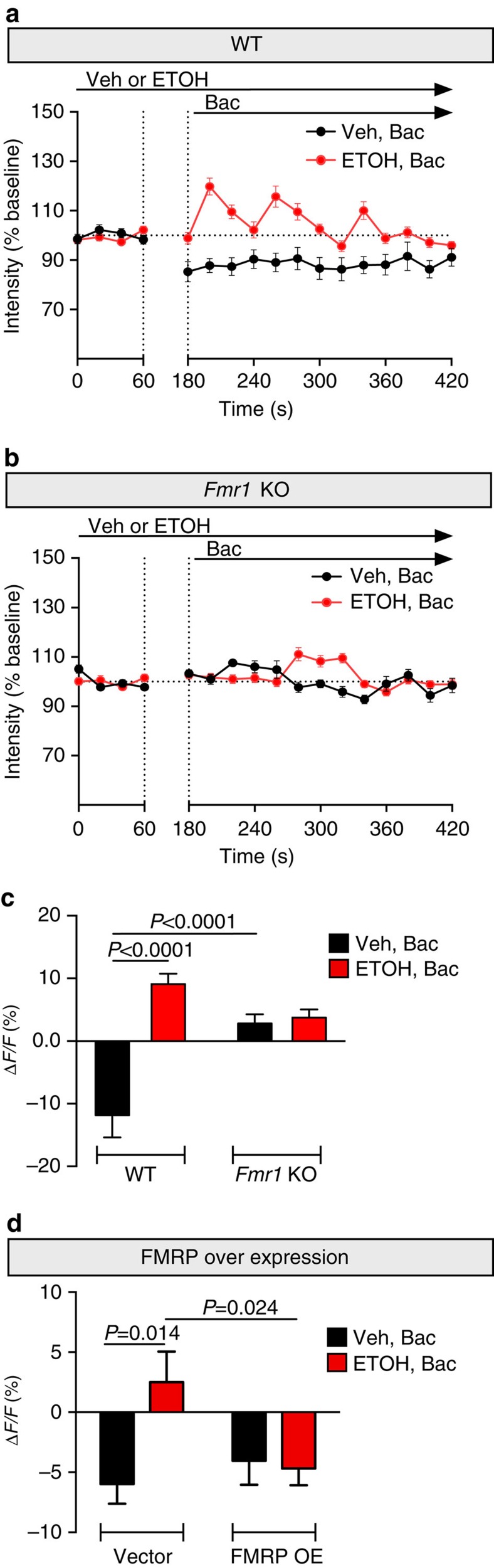
GABA_B_R plasticity and signalling is absent in *Fmr1* KO mice. (**a**,**c**) Mouse hippocampal cultured neurons were pre-treated for 2 h with either vehicle (Veh: H_2_O) or ethanol (ETOH: 30 mM). Line graphs represent the average fluorescent calcium signal in dendrites over time from (**a**) wild type (WT) and (**b**) *Fmr1* KO mice. Baseline was established for 1 min before the addition of GABA_B_R agonist baclofen (Bac: 50 μM) in vehicle- or ethanol-exposed neurons. Baclofen was allowed to equilibrate as indicated by the break between dotted lines. (**c**) Summary graph shows significant increase in dendritic calcium signal (Δ*F*/*F*) with the addition of baclofen in WT neurons pre-treated with ethanol, which was not observed in *Fmr1* KO neurons. WT: Veh+Bac= −11.82±3.55, *n*=8; ETOH+Bac=9.10±1.65, *n*=14. *Fmr1* KO: Veh+Bac=2.81±1.48, *n*=12; ETOH+Bac=3.74±1.30, *n*=12. (**d**) Dendritic calcium imaging was performed as before in hippocampal cultured neurons infected with either vector (rAAV:mSYN-tdTomato) or FMRP overexpression (rAAV:mSYN-FMRP and rAAV:mSYN-tdTomato). Ethanol-induced increase in dendritic calcium is prevented by FMRP overexpression. Vector: Veh+Bac: −6±1.6, *n*=17 dendrites; ETOH+Bac: 2.5±2.5, *n*=17 dendrites. FMRP overexpression: Veh+Bac: −4±2, *n*=11 dendrites; ETOH+Bac: −4.7±1.4, *n*=27 dendrites. Significance determined by two-way analysis of variance, followed by Tukey's multiple comparison. Values represent mean±s.e.m.

We previously demonstrated that rapid antidepressants shift GABA_B_R signalling from opening potassium channels to increasing dendritic calcium^6^. To determine whether ethanol (30 mM, 2 h) causes the same plasticity in GABA_B_R signalling, we performed fluorescence calcium imaging in cultured WT and *Fmr1* KO hippocampal neurons. A transient rise or fall in calcium in dendritic compartments can be detected using a fluorescent indicator that exhibits changes in fluorescent properties depending on the amount of bound calcium^39^. We used baclofen, a GABA_B_R agonist, to activate GABA_B_Rs in the presence or absence of ethanol. After establishing a baseline measurement, baclofen reduced dendritic calcium fluorescence in saline-treated WT neurons by ∼11%, a characteristic signature of GABA_B_R signalling increasing outward potassium conductance^8^. However, in ethanol-treated WT neurons, baclofen induced distinct calcium waves and an overall averaged increase in calcium signal of ∼9% (Fig. 7a–c and Supplementary Fig. 4a,c). These results recapitulate our previous observations with NMDAR antagonists^6^. In addition, these findings in WT mouse neurons are consistent with what we observed in rat cultured hippocampal neurons treated with ethanol or the clinically relevant rapid antidepressant Ro-25-6981 (Supplementary Fig. 5). Unexpectedly, GABA_B_R activation in saline-treated *Fmr1* KO neurons failed to reduce the calcium signal. Moreover, in ethanol-treated *Fmr1* KO neurons, GABA_B_R activation failed to increase dendritic calcium signal (Fig. 7b,c and Supplementary Fig. 4b,c). These findings suggest that the loss of FMRP in *Fmr1* KO dendrites decouples GABA_B_Rs from potassium channels. These results also suggest that the dynamic, ethanol-induced plasticity in GABA_B_R signalling, which is observed with rapid antidepressants, requires FMRP^6^.

To further substantiate that FMRP regulates ethanol-dependent GABA_B_R plasticity, we overexpressed FMRP in rat hippocampal neurons. Overexpression of FMRP did not alter the GABA_B_R activation in saline-treated neurons because baclofen reduced the dendritic calcium signal. However, in ethanol-treated neurons, overexpressing FMRP blocked the ethanol-induced GABA_B_R plasticity (Fig. 7d and Supplementary Fig. 4d,e). These results provide additional evidence that the dynamic reduction of FMRP with ethanol exposure is important for the expression of GABA_B_R plasticity.

### Antidepressant effect of ethanol on behaviour requires FMRP

**Figure 8:**
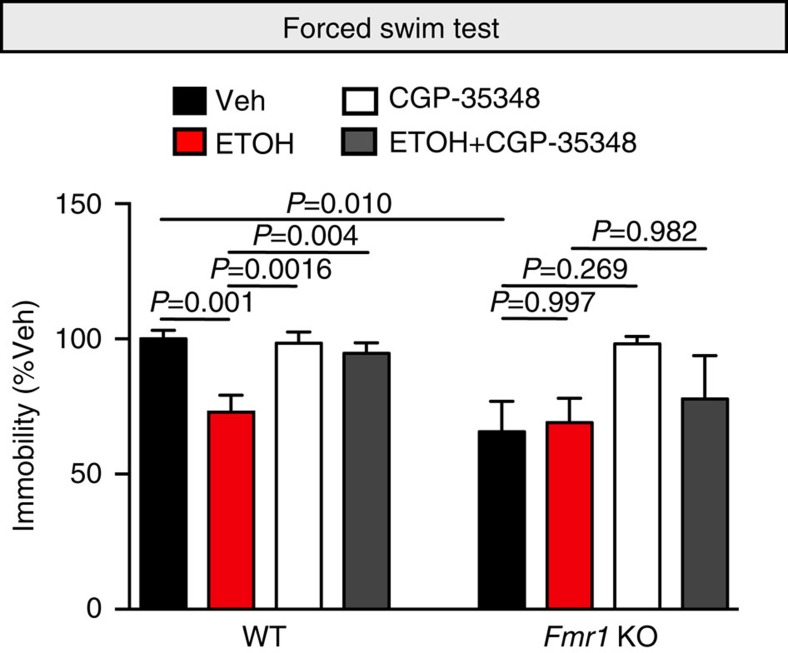
Ethanol's antidepressant effect requires GABA_B_R activation. Wild type (WT) C57BL/6 and *Fmr1* KO male mice were subjected to the forced swim test (FST) 24 h post injection of vehicle (Veh: saline), ethanol (ETOH: 2.5 g kg^−1^), CGP-35348, a GABABR antagonist (CGP: 100 mg kg^−1^) or ethanol+CGP-5348. Ethanol-induced decrease in immobility was absent in *Fmr1* KO mice. WT: Veh=100±3.19 s, *n*=9 mice; ETOH=72.97±6.23 s, *n*=7 mice; CGP-35348=98.38±4.2 s, *n*=10; ETOH+CGP-35348=94.73±3.77 s, *n*=7 mice. *Fmr1* KO: Veh=58.75±10.33 s, *n*=9; ETOH=69.02±8.99 s, *n*=3; CGP-35348=88.00±9.56 s, *n*=3; ETOH+CGP-35348=77.78±16.04 s, *n*=3. Significance determined by two-way analysis of variance Tukey's multiple comparison test. Values represent mean±s.e.m.

Since FMRP is important for ethanol-induced GABA_B_R plasticity, we examined antidepressant and anxiolytic-like effects of ethanol on behaviour in *Fmr1* KO mice. Interestingly, ethanol administration did not affect the behaviours of *Fmr1* KO mice in the splash and open field tests compared with saline-treated mice (Supplementary Fig. 6a–e). Surprisingly, the basal state of immobility in the FST in *Fmr1* KO mice is equivalent to ethanol-injected WT mice (Fig. 8). To explore this paradox, we examined the requirement of GABA_B_R activation in ethanol-induced decreases in immobility by using CGP-35348 to inhibit postsynaptic GABA_B_Rs. We previously showed that GABA_B_R antagonism blocked the antidepressant-like behaviour produced by NMDAR antagonist in the FST^6^. GABA_B_R inhibition alone did not affect the immobility of saline-injected WT mice in the FST, similar to what we observed previously (Fig. 8)^6^. CGP-35348, however, abolished the ethanol-induced antidepressant behaviour, demonstrating a requirement for GABA_B_R activation in ethanol-triggered reduction of immobility. Neither ethanol, CGP-35348, nor ethanol+CGP-35348 treatment in *Fmr1* KO mice produced immobility scores that were significantly different from saline-treated *Fmr1* KO mice. These findings collectively demonstrate that GABA_B_Rs and FMRP are necessary to elicit the ethanol-mediated antidepressant response.

## Discussion

Emerging behavioural and molecular evidence demonstrate that NMDAR antagonists act as rapid antidepressants^5^,^6^,^8^,^24^,^40^. Because it has long been speculated that individuals with major depressive disorders self-medicate with alcohol, we examined whether ethanol, which blocks NMDARs^9^, acts through the same synaptic pathways as NMDAR antagonists. Until this study, the molecular mechanisms shared by alcohol and antidepressants were unexplored. Here we provide molecular and behavioural evidence that acute alcohol exposure elicits antidepressant-like behaviours that persist up to 24 h after administration (Fig. 1), supporting the hypothesis that ethanol initiates lasting antidepressant activity. We have previously demonstrated that NMDAR inhibition by rapid antidepressants induces two key molecular changes that are responsible for the rapid antidepressant response, namely (1) an increase in GABA_B_R protein synthesis and (2) a shift in GABA_B_R function that increases dendritic calcium signalling^6^,^8^. Our current work shows that these same signature changes are produced by acute ethanol exposure (Figs 5, 6, 7).

Surface expression of functional GABA_B_Rs requires the dimerization of GABA_B_R1 and R2 subunits. Without GABA_B_R2, GABA_B_R1 is retained in the endoplasmic reticulum^32^. Our current studies show that the release of GABA_B_R2 mRNA translational repression by FMRP is necessary for the ethanol-induced increase in surface GABA_B_Rs with NMDAR blockade (Figs 4, 6 and 8; Supplementary Fig. 3). Reduction of FMRP, as seen in animal models of FXS, is associated with elevated protein synthesis of target mRNAs^16^. While we have demonstrated that FMRP associates with GABA_B_R1 and R2 mRNAs, the loss of FMRP has a profound effect on GABA_B_R2 protein expression in dendrites. Constitutive loss of FMRP, as observed in many of its targets, abrogates stimulus-dependent mRNA transport and translation of target mRNAs^41^,^42^,^43^,^44^. We show that the ethanol-induced increase in GABA_B_R2 protein is also absent in *Fmr1* KO mice.

The question of why GABA_B_R2 is uniquely affected by acute ethanol and its dependence on reduced FMRP levels is intriguing. Both GABA_B_R1 and GABA_B_R2 mRNAs were detected in the FMRP RIP (Fig. 3). Interestingly, *in vivo* FMRP influences GABA_B_R1 expression to a lesser extent compared with GABA_B_R2, suggesting that FMRP may act in concert with other repressors such as microRNAs to tightly regulate GABA_B_R1 expression^45^. Moreover, our immunostaining and protein synthesis assays suggest that the increased protein levels of GABA_B_R1 are not due to new protein synthesis in the dendrites, even in *Fmr1* KO mice (Figs 5 and 6). These results indicate that the overall increase in synaptic GABA_B_R1 expression in *Fmr1* KO mice may be due to an increase in presynaptic GABA_B_R1 expression, either through protein synthesis or increased protein stability. Notably, FMRP has been localized to axons and presynaptic terminals^46^,^47^. Further exploration into presynaptic and postsynaptic GABA_B_R expression and function in AUD and FXS is warranted.

The ‘GABA hypoinhibition theory' posits that loss of inhibition is a leading cause in many of the neurological symptoms observed in FXS^48^. While studies showing reduced inhibition in models of FXS have focused on decreased expression of GABA_A_R subunit mRNA and protein^49^, GABA_B_R protein expression and dendritic signalling has not been explored. Interestingly, the GABA_B_R agonist baclofen has shown promise in treating FXS. (R)-baclofen administration *in vitro* corrects the elevated basal protein synthesis normally seen in *Fmr1* KO mice, and rescues synaptic abnormalities such as increased spine density^50^. In addition, baclofen administration reduced symptoms related to FXS in *Fmr1* KO mice^50^,^51^. Recent studies have shown that the excitatory drive to fast spiking inhibitory neurons is reduced in the cortex of *Fmr1* KO mice^52^. Our data expand on these findings by suggesting that postsynaptic GABA_B_R coupling to inwardly rectifying G protein-coupled potassium channels (GIRK) is absent in *Fmr1* KO neurons (Fig. 7). Collectively, these results may imply that the therapeutic effects of baclofen in the *Fmr1* KO mouse may be due to the activation of presynaptic GABA_B_ receptors that may in turn reduce glutamate release and reduce hyperactive metabatropic glutamate receptor mGluR signalling in the hippocampus^53^.

Changes in gene expression and protein synthesis are essential for normal neuroplasticity, but these crucial processes are dysregulated by drug addiction^18^,^54^. Several lines of evidence support parallel changes in GABA_B_R mRNA translation/signalling as a result of NMDAR blockade that may be critical for alcohol actions. First, NMDAR antagonists mimic some effects of ethanol in humans^55^, suggesting common biochemical/electrophysiological signalling pathway(s). Second, changes in GABA_B_R2 brain gene expression correlates with lifetime alcohol consumption, supporting a role for altered GABA_B_R signalling in AUD^56^. Third, although controversial, the GABA_B_R agonist baclofen may decrease alcohol consumption in some alcoholics^57^. In summary, our data define a common molecular paradigm for alcohol and rapid antidepressants, and identify a mechanism for the initial antidepressant effects of alcohol. A shift in GABA_B_R signalling is observed with both rapid antidepressants and acute ethanol treatment, which may provide insight into the molecular basis for the high comorbidity between major depressive disorder and AUD.

*Note added in proof:* Spencer *et al*. found fmrp mediated changes ion channels with chronic ethanol exposure^58^.

## Methods

### Cell culture

Primary hippocampal neurons were prepared as previously described by Niere *et al*.^43^. Briefly, hippocampi were extracted from postnatal day 1–3 Sprague–Dawley rat pups, WT C57BL/6 mouse pups, or *Fmr1*-knockout (*Fmr1* KO) mouse pups on a C57BL/6 background. The tissue was dissociated and plated in neurobasal A medium supplemented with B27, glutamine, and 1% fetal bovine serum. Cultures were plated at a density of ∼100,000 cells per 12 mm on glass coverslips that had been coated overnight with 50 μg ml^−1^ poly-D-lysine and 25 μg ml^−1^ laminin in borate buffer. Cultures were fed after 1 day *in vitro* (DIV), and media was replaced approximately once a week with either fresh rat culture media (neurobasal A supplemented with B27, glutamine and 3 μM AraC) or fresh mouse culture media (glial-conditioned media with 3 μM AraC) until cultures were used at DIV 14–21.

### *In vitro* pharmacology

Primary hippocampal neurons were treated in ethanol vapour chambers according to a method adapted from Chandler *et al*.^59^. Ethanol vapour chambers were prepared by placing a reservoir of 31.5 mM ethanol (105% of the desired ethanol concentration, that is, 30 mM) in a plastic container with 24-well culture plates containing neuronal cultures in which 30 mM ethanol was added to the culture media. Chambers were filled with 95% O_2_/5% CO_2_ and cultures were incubated for 2 h at 37 °C. Cultures treated with vehicle (H_2_O) were incubated in the same manner but in the absence of ethanol. For calcium imaging, ethanol was added directly to HEPES-based artificial cerebral spinal fluid (ACSF in mM: 100 NaCl, 10 HEPES (pH 7.4), 3 KCl, 2 CaCl_2_, 1 MgCl_2_, 10 glucose) that was adjusted to match the osmolarity of cell culture media for live imaging. For GABA_B_R activation neurons were treated with (R)-baclofen (50 μM, Tocris). For Figs 5 and 6, cultured hippocampal neurons were pre-treated with cycloheximide (50 μM, Tocris) for 10 min before ethanol treatment. For Supplementary Fig. 3, neurons were treated with Ro-25-6981 (10 μM, Tocris) or Veh (H_2_O) for 2 h. All cultures were treated at 14–21 DIV. Following treatment, cultures were immediately fixed or live imaged.

### *In vivo* pharmacology

Male C57BL/6 mice (Charles Rivers) or *Fmr1-*knockout (*Fmr1* KO) mice on a C57BL/6 background (at least 7 weeks old) were given i.p. injections of either 200 μl saline or 2.5 g kg^−1^ ethanol (in a volume of 200 μl saline)^23^. For Fig. 8, CGP-35348 (100 mg kg^−1^) was i.p. injected with or without ethanol (in a volume of 200 μl saline). All animals were housed four mice per cage according to genotype. All treatments were administered to one mouse per home cage. At the time of drug treatment, animals were coded by number. During the behavioural tasks, animal performance was video recorded, and then later blindly scored. In certain tasks (for example, open field), the animals were scored by a computer program and blinding was not necessary during that process. For western blots the hippocampi were isolated 30 min post injection and flash-frozen. All experiments were carried out in accordance with the National Institutes of Health's Guide for the Care and Use of Laboratory Animals and approved by the UT-Austin Institutional Animal Care and Use Committee (IACUC).

### Immunofluorescence

Primary neuronal cultures on glass coverslips were fixed with 4% paraformaldehyde (PFA) at room temperature for 20 min, washed three times with phosphate-buffered saline (PBS) and permeabilized in 0.25% Triton X-100 in PBS for 5 min. For FMRP staining, neurons were fixed and permeabilized in 100% methanol at −20 °C for 10 min. Neurons were washed three times in PBS and then blocked (10% normal goat serum in PBS) for 30 min at room temperature. Primary antibodies were incubated in blocking buffer at 4 °C overnight. Neurons were washed three times for 10 min with PBS, and then incubated in secondary antibody in blocking buffer for 1 h at room temperature, and washed three times for 10 min with PBS before mounting in Fluoromount with DAPI (SouthernBiotech, 0100-20) as outlined in Sosanya *et al*.^60^. Surface staining was performed similarly to Workman *et al*.^6^. Neurons were first fixed in 4% PFA for 10 min on ice, washed three times in PBS, blocked with 3% normal goat serum, and then incubated in primary surface antibody in 3% blocking buffer overnight at 4 °C. Following primary surface antibody incubation, neurons were washed six times for 10 min each in PBS, then permeabilized with 0.25% Triton X-100 in PBS for 5 min, followed by three washes for 10 min each in PBS, and again incubated in primary total antibody in 3% blocking buffer overnight at 4 °C. Neurons were washed three times for 10 min each with PBS, and then incubated in secondary antibody in 3% blocking buffer for 1 h at room temperature, and finally washed four times for 10 min with PBS before mounting in Fluoromount with DAPI to slides. The primary antibodies used were: Total GABA_B_R1 (1/50 dilution; Santa Cruz, sc-14006), Surface GABA_B_R1 (1/200 dilution; Abcam, ab55051), GABA_B_R2 (1/100; Neuromab 75-124), FMRP (1/500 dilution; Abcam ab17722), MAP2 (1/2000 dilution; Abcam ab5392), GFP (1/1,000 dilution; Aves, GFP-1020). Secondary antibodies included: Alexa488, 555 and 647 developed in goat (1/500 dilution; Life Technologies, A-11039, A-11017, A-31621, A-21430, A-21449, A-21240 and A-21237).

### Adeno-associated viral vectors

The FMRP and tdTomato coding sequences were cloned into separate adeno-associated viral (AAV) vectors containing a mouse synapsin promoter, a woodchuck post-transcriptional regulatory element and SV40 poly-adenylation sequence between flanking AAV2 inverted terminal repeats. rAAVs were assembled using a modified helper-free system (Stratagene) as serotype 2/1 (*rep*/*cap* genes) viruses, and collected and purified over sequential caesium chloride gradients as previously described^61^. Viral titres were >1 × 10^9^ infectious particles per microlitre. For FMRP and&#8232;tdTomato co-infections, rAAV:mSYN-FMRP and rAAV:mSYN-tdTomato were mixed at a ratio of 4:1. One microlitre of the resulting rAAV mix was used per coverslip of primary cultured neurons. Imaging was performed ∼1 week after infection.

### Live calcium imaging

Dissociated hippocampal cultures were prepared from WT and *Fmr1* KO mice as described^43^. Neurons at 14–21 DIV were used for live calcium imaging. Neurons were treated as outlined in *in vitro* pharmacology above. Before imaging, cells were incubated in ACSF with Oregon Green 488 BAPTA-1 AM (OGB, 200 μM; 30 min; 37 °C; ThermoFisher) as described^6^. After OGB incubation, cells were transferred to fresh ACSF (37 °C) for imaging (1 frame per 20 s). Baseline calcium signal was imaged (1 min), after which (R)-baclofen (50 μM, Tocris) or vehicle (H_2_O) was added. For ethanol-treated cells, the neurons were incubated with OGB and imaged in ACSF containing ethanol (30 mM). Neurons were imaged for 800 s at room temperature. Quantification of the calcium signal was performed using Metamorph (Molecular Devices) as described^6^. Briefly, dendritic regions of interest (ROI) that were at least 5 μm from the soma were analysed. The mean intensity values for each ROI at each time were averaged as baseline (*F*_0_). The ROI intensity values obtained at each time point after the addition of baclofen or vehicle were averaged (*F*). The equation, Δ*F*/*F*=((*F*−*F*_0_)/*F*_0_), was used to measure the change in signal and data were plotted as a percentage of the baseline.

### BONCAT-PLA

BONCAT-PLA was performed using Click-it Metabolic Labeling azidohomoalaine (AHA), Biotin-Alkyne and Click-iT Reaction buffer kit (Life Technologies). Proximity ligation assay (PLA) was performed using Duolink kit (Duolink, Sigma)^38^. Briefly, primary hippocampal neuronal cultures were incubated in a methionine-free ACSF media for 30 min. AHA was then added to the media just before neurons were treated with ethanol for 2 h as previously described. Neurons were fixed in 4% PFA for 15 min, washed two times for 5 min with 3% bovine serum albumin (BSA) in PBS, followed by permeabilization with 0.25% Triton X-100 in PBS for 15 min, and washed as before. Neurons were incubated for 30 min at room temperature in Cell Buffer Additive/Click-it Cocktail according to manufacturer directions. Neurons were washed as before and then blocked and incubated with primary antibody as previously described. Next, neurons were incubated in the appropriate PLA probes diluted in blocking buffer and secondary antibody at 37 °C for 1 h. Neurons were washed in RT Buffer A two times for 5 min, and incubated in ligation solution at 37 °C for 30 min, and washed again in Buffer A. Neurons were incubated in amplification solution at 37 °C for 2–3 h, followed by washing in RT Buffer B two times for 10 min and 1% buffer B for 1 min. Last, neurons were mounted to slides in Duolink mounting media for imaging. Primary and secondary antibodies included: GABA_B_R1 (1/50 dilution; Santa Cruz, sc-14006), GABA_B_R2 (1/100; Neuromab 75-124), MAP2 (1/2,000 dilution; Abcam, ab5392), biotin/α-rabbit (1/500; Sigma, SAB3700857), Alexa488 (1/500; Life Technologies, A-11039). PLA probes used: Rabbit Minus (1/5; Duolink, 82005), Mouse Plus (1/5; Duolink, 82001).

### Microscopy and analysis

Images were acquired with a Leica SP5 confocal microscope under a × 63 oil immersion lens for fixed tissue or a × 63 water immersion lens for live imaging. Max projected images were used for immunostaining analysis from 10 μm Z-stacks of 1,024 × 1,024 pixels obtained using a 400-Hz scan rate^60^. For each experiment, all images were collected using the same settings. Fixed images were analysed using NIH imaging software ImageJ, and live imaging quantification was performed with Metamorph (Molecular Devices, Sunnyvale, CA). Background signal was determined by shifting the ROI adjacent to the dendrite being traced, but void of all processes. Dendritic signal was background subtracted and averaged every 10 μm using a customized R script.

### Western blot analysis

Protein was isolated from hippocampal synaptoneurosomes prepared from male mice age 7–8 weeks treated with ethanol or vehicle as previously described. synaptoneurosomes were prepared by homogenizing hippocampal tissue in homogenization buffer (20 mM HEPES pH 7.4, 5 μM EDTA pH 8.0, and protease inhibitor cocktail). Homogenate was filtered through a 100-μm nylon filter followed by a 5-μm filter, and centrifuged at 14,000*g* for 20 min at 4 °C (ref. 62). The pellet was resuspended in RIPA buffer (150 mM NaCl; 10 mM Tris pH 7.4, 0.1% SDS, 1% Triton X-100, 1% deoxycholate, 5 mM EDTA and protease inhibitor cocktail) and centrifuged for 10 min. The supernatant was used for western blot analysis. Protein was separated on a 4–20% gradient SDS–polyacrylamide gel. The gel was then transferred to a nitrocellulose membrane, blocked in 5% non-fat dry milk in tris-buffered saline and 0.1% Tween20 (TBST) for 1 h, and incubated with primary antibody in blocking buffer overnight at 4 °C. The blot was washed in TBST three times for 10 min each, incubated in secondary antibody for 1 h, and washed as before. Blots were imaged using a LICOR Odyssey imaging system, and ImageJ was used for densitometry analysis. Representative images are pseudocoloured with black (lowest intensity at 0 pixels) to red (highest intensity at 255 pixels) using LICOR Image Studio software. Primary antibodies used consisted of: GABA_B_R1 (1/400 dilution; Santa Cruz, sc-14006), GABA_B_R2 (1/800; Neuromab 75-124), alpha-Tubulin (1/2,000 dilution; Sigma, T6074). Secondary antibodies included: anti-mouse-IR-Dye 800 (1/5,000 dilution excluding tubulin at 1/10,000 dilution; LICOR, 96-32210) and anti-rabbit Alexa680 (1/5,000 dilution; Invitrogen, A-21084).

### RNA immunoprecipitations

Cortices from 6-week-old C57BL/6 and *Fmr1* KO male mice were collected and flash-frozen on dry ice. RIP was performed by modified method of Jain *et al*. and Keene *et al*.^63^,^64^. Tissue was homogenized and lysed with a cordless pestle motor and disposable pellet mixers (VWR) in polysome lysis buffer (10 mM HEPES pH 7.0, 100 mM KCl, 25 mM EDTA, 5 mM MgCl_2_, 1 mM DTT, 0.5% NP-40) in a 1:1 tissue-buffer ratio. RNaseOUT (Thermo) and protease/phosphatase inhibitors (Halt Protease and Phosphatase Cocktail, Pierce Biotechnology) were freshly added to samples. Samples were rotated for 10 min at 4 °C to induce swelling and then flash-frozen on dry ice. Samples were thawed by holding between fingers at room temperature to lyse and nuclei were pelleted at 3,000*g* for 10 min. Lysates obtained above were pre-cleared by adding 50 μl of washed magnetic bead slurry (Protein A Dynabeads, Thermo) and rotating for 30 min at 4 °C. To bind the antibody to the beads, 50 μl of magnetic beads slurry was washed and then resuspended in eight volumes of NT-2 buffer (50 mM Tris-HCl, pH 7.4, 150 mM NaCl, 1 mM MgCl_2_, 1 mM DTT, 0.05% NP-40 with RNaseOUT/protease and phosphatase inhibitors added fresh)+5% BSA. Ten micrograms of either FMRP (Abcam, ab17722) or IgG (Santa Cruz Biotechnologies, sc-2027) antibodies was added to the beads and rotated for 10 min at room temperature. Antibody-bound beads were washed four times with ice-cold NT-2 buffer. For the immunoprecipitation, 4.5 mg of protein from pre-cleared lysates was added to an RNase-free microcentrifuge tube containing the antibody-bound beads. Input collected at this step for downstream analysis was either 1% of the final pre-cleared lysate volume in the immunoprecipitation reaction (for immunoblotting) or 10% of the final pre-cleared lysate volume (to normalize in qPCR). The antibody–bead-lysate mixture was then diluted at a ratio of 1:5 with NET-2 buffer (20 mM EDTA pH 8.0, and 1 mM DTT in NT-2 buffer; RNaseOUT and protease/phosphatase inhibitors added fresh) and rotated for 1 h at room temperature. Beads were quickly washed six times in ice-cold NT-2 buffer and immediately resuspended in 350 μl TRI Reagent Solution (Ambion) for 10 min at room temperature. Beads were pelleted and the supernatant was removed and resuspended in 350 μl of absolute ethanol. RNA was extracted by applying ethanol-resuspended samples to spin column from the Direct-zol RNA MiniPrep Kit (Zymogen) according to the manufacturer's instructions. Eluted RNA (25 μl) was DNase treated using the TURBO DNA-free kit (Thermo).

### cDNA synthesis and quantitative real-time PCR

DNase-treated RNA samples were reverse-transcribed to complementary DNA using the iScript cDNA Synthesis Kit (Bio-Rad) in a 20 μl volume according to the manufacturer's instructions. Quantitative real-time PCR (qRT–PCR) was performed in 20 μl reaction volume using the iQ SYBR Green Supermix (Bio-Rad) and primers for GABA_B_R1 (GeneCopoeia, MQP031832), GABA_B_R2 (GeneCopoeia, MQP026008), CaMKIIα (GeneCopoeia, MQP028785), and Cacna2δ2 (GeneCopoeia, MQP032309). qRT–PCR was run with the following protocol: 95 °C for 10 min, 40 cycles of 95 °C for 15 sec followed by 60 °C for 1 min, 95 °C for 1 min, and 55 °C for 1 min. Relative fold-enrichment was determined by the equation ΔΔCt=2^−(Ct FMRP RIP−Ct IgG RIP)−(Ct FMRP input−Ct IgG input)^ (ref. 65).

### Forced swim test

Male mice were tested in the forced swim test 24 h post injection as described previously^6^,^8^. Mice were individually placed into a cylinder containing 3 l of water (25 °C) for 6 min. Each session was video recorded and the last 4 min of the sessions were later scored blindly for immobility. Animals were scored for escape-directed behaviours. The water was replaced between animals. Experiments were repeated by three independent experimenters. Data were normalized by experimenter. Power analysis was performed in R Programming^66^ to predict sample size for all behavioural tests. This sample size was used as a guideline for the WT animals, however since transgenic animals were used, the exact sample size for each group may not have been possible. Transgenic animal sample size was as close as possible to that which was calculated, due to limitations of litter size.

### Open field

Twenty-four hours after animals were injected i.p. with either saline or ethanol, they were studied in the open field test similar to Treit *et al*.^31^. Mice were individually placed in a 40 cm × 40 cm × 35 cm arena with opaque walls. Each test session lasted 30 min under 85 lux illumination. Sessions were video recorded and analysed via ANY-Maze (Stoelting, Wood Dale, IL). Mice were considered to be in the centre of the maze if they entered a 18.5 cm × 18.5 cm area in the centre of the apparatus. Mice were returned to their home cage at the end of the test session, and the arena was wiped down with 70% ethanol before the start of each run.

### Splash test

Two and a half hours after open field testing, animals underwent the splash test^27^,^28^. Cage mates were moved from their home cage to a holding cage, and each animal was individually tested in its home cage. Two-hundred microlitres of 10% sucrose was applied to the dorsal fur of the mouse. Mice were monitored and video recorded for 5 min and then moved to a different holding cage. Videos were later scored blindly for latency to initiate grooming and for total time spent grooming. Grooming behaviour included licking, grooming with forepaws, and scratching.

### Statistical analysis

Power analysis was performed in R Programming^66^ to predict sample size. Prism software (GraphPad) was used for all statistical analyses. Statistical comparisons were made using one-way analysis of variance (ANOVA), two-way ANOVA, Student's *t*-test or *χ*^2^-test with Yates. Outliers were determined using Grubbs' test (α=0.05). All data are expressed as mean±s.e.m.

### Data availability

All relevant data are available from the authors upon request. Accession numbers for deposited date used in Fig. 4a: GSE26809 (ref. 35) and SRA029279 (ref. 37).

## Additional information

**How to cite this article:** Wolfe, S. A. *et al*. FMRP regulates an ethanol-dependent shift in GABA_B_R function and expression with rapid antidepressant properties. *Nat. Commun.* 7:12867 doi: 10.1038/ncomms12867 (2016).

## Supplementary Material

Supplementary Information Supplementary Figures 1-7.
