# Supplementary material for: FMRP regulates an ethanol-dependent shift in GABABR function and expression with rapid antidepressant properties
Source: Nat Commun. 2016 Sep 26;7:12867. doi: 10.1038/ncomms12867 (PMC5052688; doi:10.1038/ncomms12867)
Supplement: Supplementary Information — Supplementary Figures 1-7. [file ncomms12867-s1.pdf]

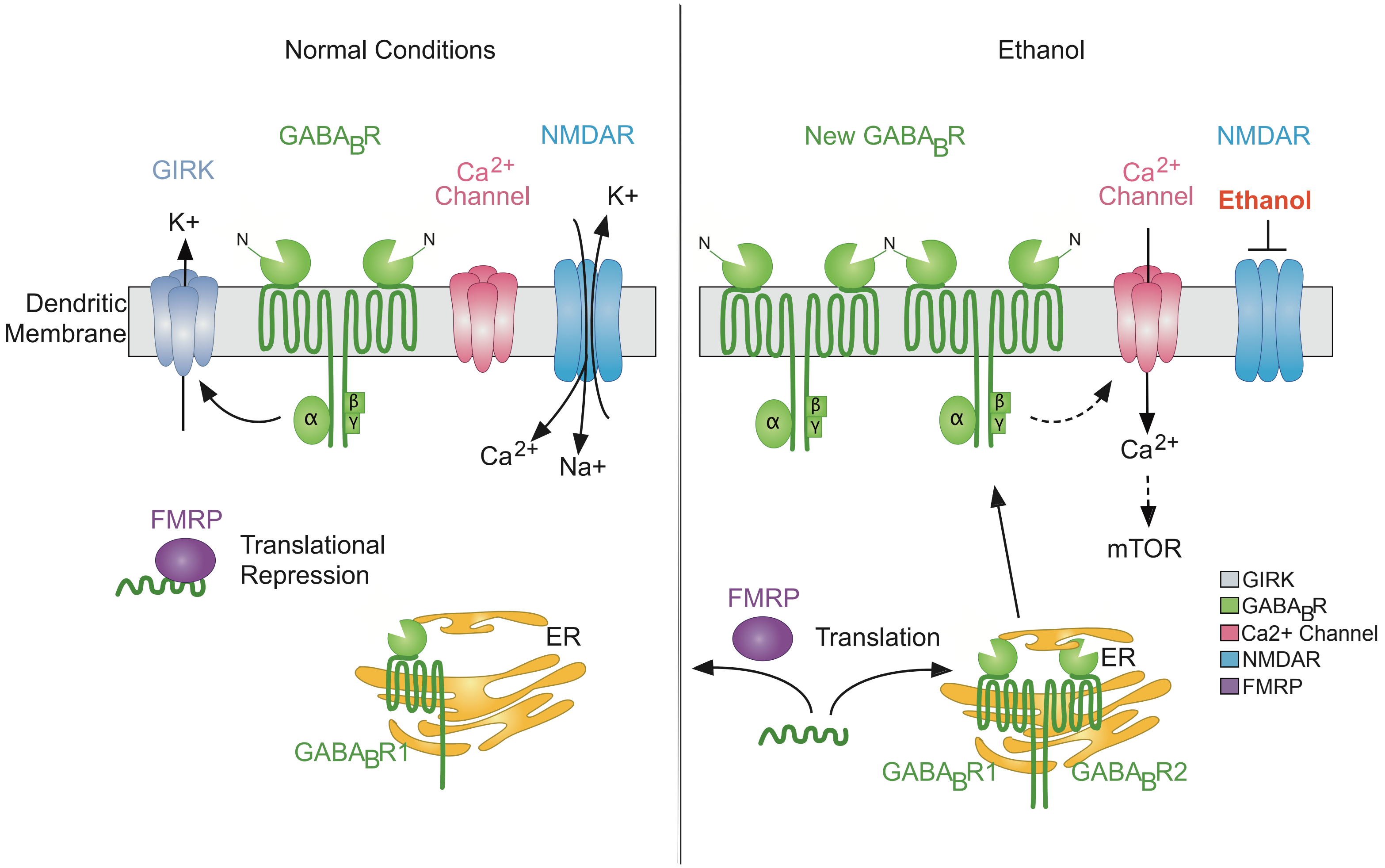


**Supplementary Figure 1. Working model of ethanol-induced GABA$_B$R plasticity**

Under normal conditions (on left) FMRP represses translation of target mRNA (e.g., GABA$_B$R2), and GABA$_B$R signaling is inhibitory via activation of G-protein inwardly rectifying potassium channels (Kir3/GIRK) in dendrites. Ethanol exposure (on right) inhibits NMDARs, causing FMRP to release GABA$_B$R2 mRNA, allowing for its translation. Newly synthesized GABA$_B$R2 assembles as a functional heterodimer with GABA$_B$R1 and is transported to the dendritic membrane. Upon activation, new surface GABA$_B$Rs facilitate calcium channel activity and may activate mTOR.

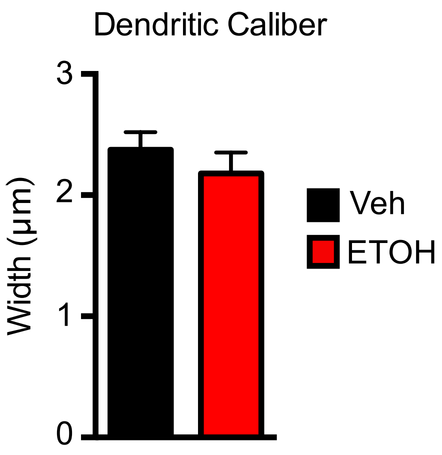


**Supplementary Figure 2. Dendritic caliber is unaffected by acute ethanol**

The width of cultured hippocampal dendrites proximal to the soma was measured using ImageJ software, and no significant changes in dendrite caliber were observed. Veh=2.38 ± 0.14 μm, n=28 dendrites; ETOH=2.18 ± 0.17 μm, n=38 dendrites; values represent mean ± SEM.

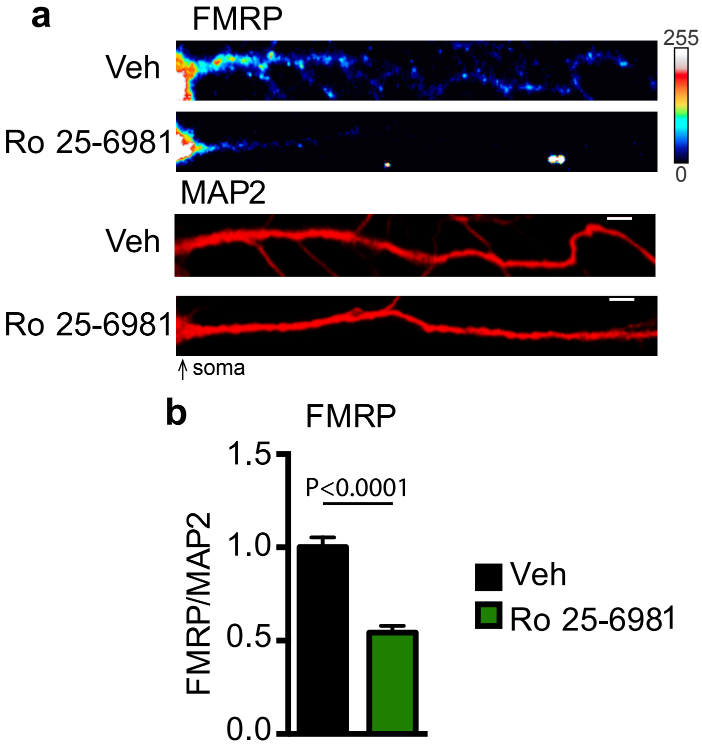


**Supplementary Figure 3. Ro 25-6981 reduces dendritic expression of FMRP**

(a) Representative immunofluorescent images and (b) summary graph of dendrites from cultured hippocampal neurons treated with Ro 25-6981 (10 μM, 2 hours) showing decreased dendritic FMRP expression (0.54 ± 0.04, n=40 dendrites) compared to vehicle (Veh)-treated neurons ($H_2O$, 2 hours; 1.00 ± 0.05, n=49 dendrites) normalized to MAP2. Significance determined by Student's t-test. Values represent mean ± SEM. Scale bar=5 μm.

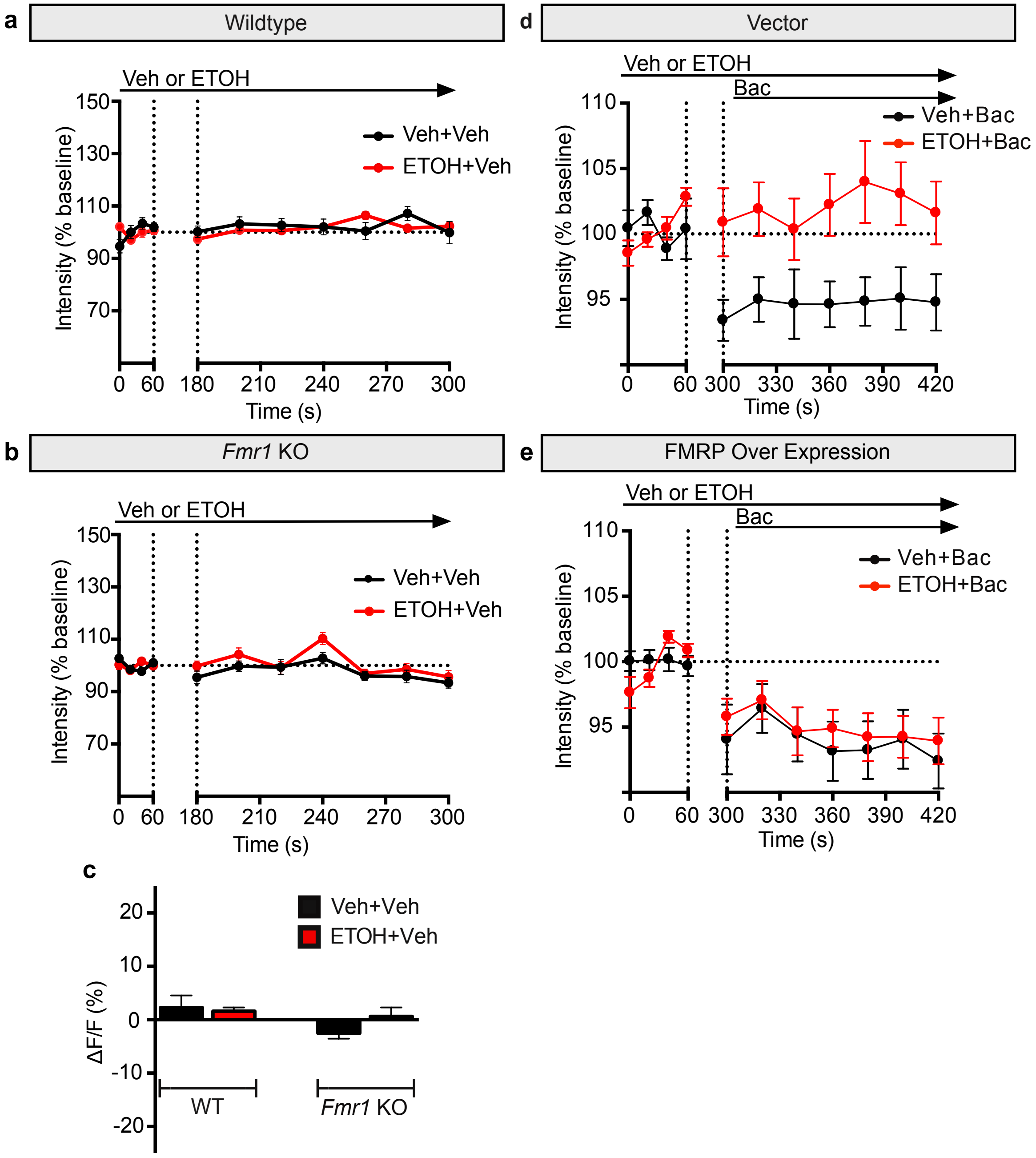


**Supplementary Figure 4. Excess FMRP represses ethanol-induced GABA$_B$R plasticity**

(a-c) Mouse hippocampal cultured neurons were pretreated for 2 hours with vehicle (Veh: $H_2O$) or ethanol (ETOH: 30 mM). Line graphs represent the average fluorescent calcium signal over time in dendrites from (a) wildtype and (b) *Fmr1* KO mice. Baseline was established for 1 minute before the addition of vehicle and equilibrated as indicated by the break between dotted lines. (c) Summary graph shows that calcium remains at baseline in dendrites imaged (ΔF/F) in wildtype (WT) and *Fmr1* KO neurons treated with vehicle or ethanol. WT: Veh+Veh=2.25 ± 2.29, n=14; ETOH+Veh=1.60 ± 0.71, n=24. *Fmr1* KO : Veh+Veh=-2.50 ± 1.05, n=12; ETOH+Veh=0.64 ± 1.68, n=12. Related to Figure 7a-c. (d-e) Dendritic calcium imaging in hippocampal cultured neurons infected with vector (rAAV:mSYN-tdTomato) or showing FMRP overexpression (rAAV:mSYN-FMRP and rAAV:mSYN-tdTomato). Line graph illustrates the average fluorescent calcium signal in (d) vector and (e) FMPR overexpressing neurons pre-treated for 2 hours with vehicle or ethanol. Ethanol-induced increase in dendritic calcium is blocked by FMRP overexpression. Related to Figure 7d. Significance determined by two-way ANOVA and Tukey's multiple comparison test. Values represent mean ± SEM.

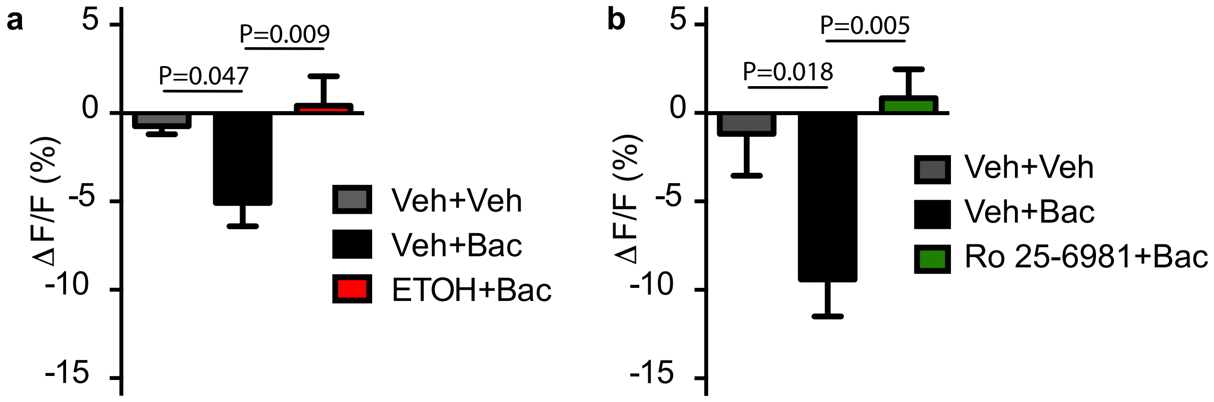


**Supplementary Figure 5. $GABA_B$R elevates dendritic $Ca^{2+}$ with ethanol and Ro 25-6981**

Calcium imaging in dendrites from rat hippocampal neurons. (a) Summary graph of ΔF/F of dendritic calcium in vehicle- (Veh: $H_2O$) and ethanol- (ETOH: 30 mM) treated neurons for 2 hours with the addition of vehicle or baclofen (Bac, 50 µM). ΔF/F between vehicle and ethanol do not significantly change relative to baseline. Baclofen decreases dendritic calcium in vehicle-treated neurons but not in ethanol-treated neurons. Veh+Veh=- 0.007 ± 0.004, n=11; Veh+Bac=-0.05 ± 0.01, n=11; ETOH+Bac=0.004 ± 0.02, n=12. Significance determined by one-way ANOVA with Dunnett's multiple comparison test. Values represent mean ± SEM. (b) Summary graph of dendritic calcium in neurons pre-treated with either vehicle (Veh: H2O, 2 hours) or Ro 25-6981 (Ro; 10 µM, 2 hours) in the presence of vehicle or baclofen (Bac, 50 µM). Baclofen reduces the calcium signal in vehicle-treated neurons but produces no significant change over baseline in the Ro 25-6981-treated neurons. Veh+Veh=-0.01 ± 0.02, n=10; Veh+Bac=-0.09 ± 0.02, n=9; Ro 25-698+Bac=0.01 ± 0.02, n=8. Significance determined by one-way ANOVA and Dunnett's multiple comparison test. Values represent mean ± SEM.

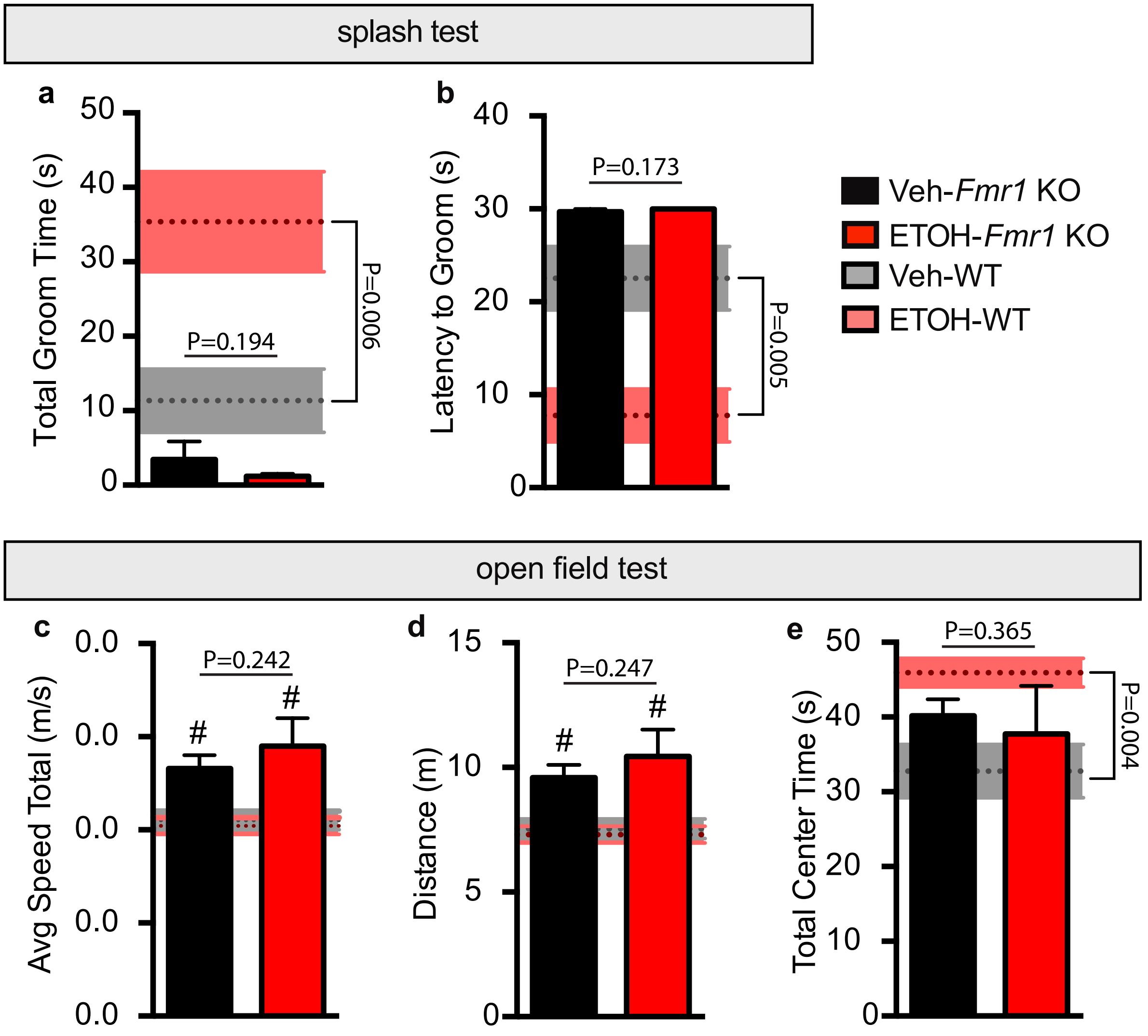


**Supplementary Figure 6. Ethanol's antidepressant effect is absent in *Fmr1* KO**

*Fmr1* KO mice subjected to the splash test displayed no behavioral differences in (a) groom time or (b) latency to initiate grooming 24 hours post-ethanol (ETOH: 2.5 g $kg^{-1}$, i.p.) compared to vehicle (Veh; saline, i.p.) treatment. Data from wildtype (WT) C57BL/6 mice from Fig. 1b and c are shown as the mean (horizontal dotted lines) ± SEM (horizontal shaded area in v=Veh=grey or ETOH=pink). Data for *Fmr1* KO as indicated in bar graph (Veh=black or ETOH=red): Total groom time: Veh=3.44 ± 2.43 s, n=5; ETOH=1.20 ± 0.32 s, n=5. Latency to groom: Veh=297.0 ± 3.05 s, n=5; ETOH=300 ± 0.0 s, n=5. (c-e) Total center time, speed, and distance were measured in the open field test 24 hours post-injection in *Fmr1* KO mice. Ethanol-treated (2.5 g $kg^{-1}$, i.p.) *Fmr1* KO mice lacked the ethanol-induced increase in center time seen in WT mice (data taken from Fig. 1b and c). Speed and distance traveled of *Fmr1* KO mice were unaffected by ethanol treatment, but both measures were increased compared to WT due to hyperexcitability in *Fmr1* KO mice. Total center time: Veh=401.6 ± 22.01 s, n=6; ETOH=377.4 ± 64.24 s, n=6. Average speed: Veh=0.05 ± 0.003 m/s, n=6; ETOH=0.06 ± 0.006 m/s, n=6. Total distance: Veh=95.89 ± 5.24 m, n=6; ETOH=104.4 ± 10.79 m, n=6. Significance determined by one-tailed t-test. Significance between genotypes designated by #. Average speed total: WT-Veh vs. KO-Veh P=0.006, WT-ETOH vs. KO-ETOH P=0.010. Distance: WT-Veh vs. KO-Veh P=0.005, WT-ETOH vs. KO-ETOH P=0.009. Values represent mean ± SEM.

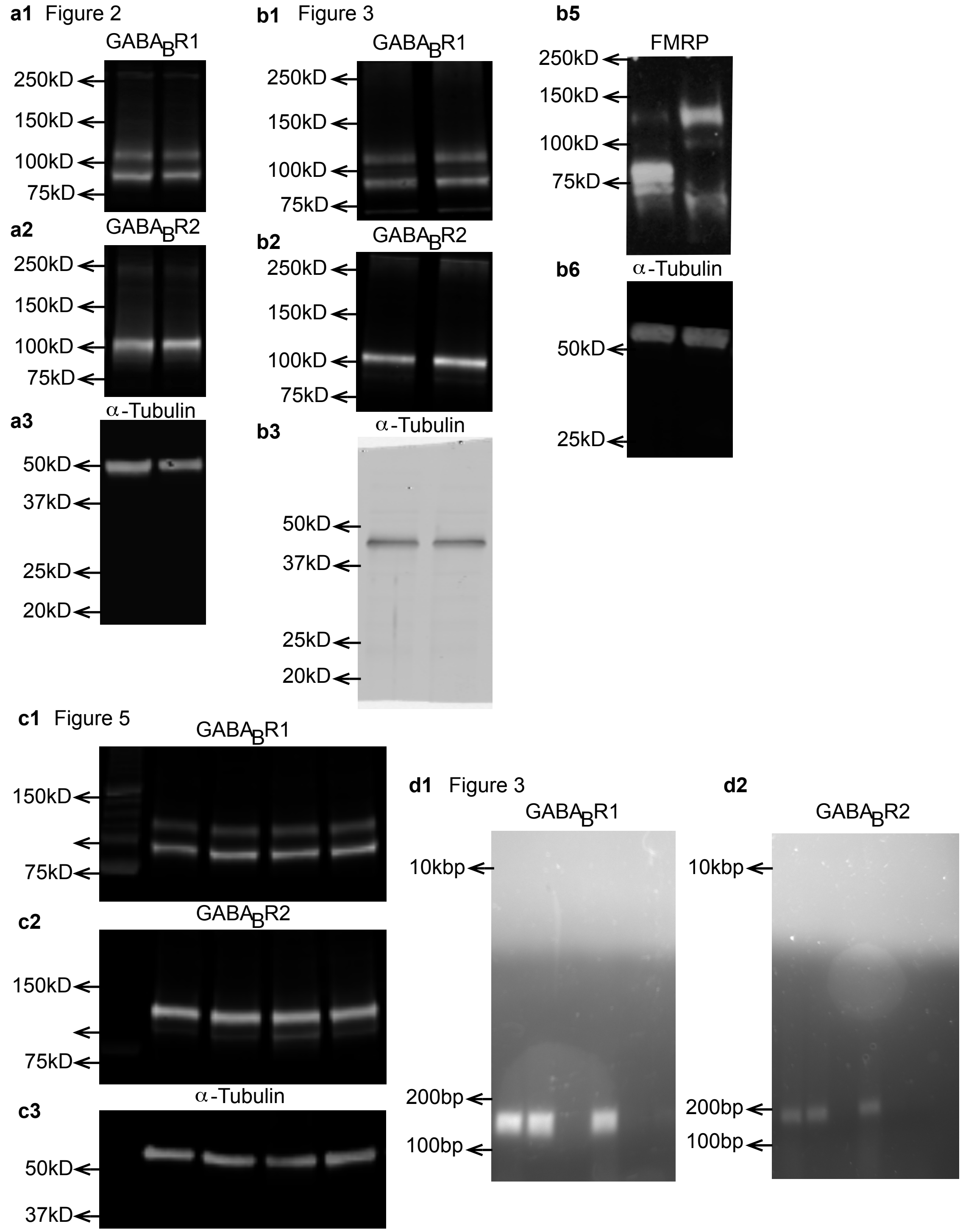


**Supplementary Figure 7. Full uncropped images for representative blots**

Full Western blots for all representative images are shown. Blots were cut to optimize staining. (a) Western blot for (a1) $GABA_BR1$, (a2) $GABA_BR2$, and corresponding (a3) α-Tubulin are shown for Figure 2. (a1-3) Blot was probed for (a1) rabbit anti-$GABA_BR1$ and (a2) mouse anti-$GABA_BR2$ simultaneously and imaged with Licor Odyssey imaging system. Prior to Western blot membrane was cut just below 75kD and probed for (a3) α-Tubulin. (b) Western blot of (b1) $GABA_BR1$, (b2) $GABA_BR2$, and corresponding (b3) α-Tubulin; and (b5) FMRP and corresponding (b4) α-Tubulin are shown for Figure 3. (b1-3) Blot was probed for (b1) rabbit anti-$GABA_BR1$ and (b2) mouse anti-$GABA_BR2$ simultaneously and was cut just below 75kD and probed for (b3) α-Tubulin. (b5-6) Western blot was probed for (b5) FMRP and cut and probed below 75kD for (b6) α-Tubulin. (c) Western blot for $GABA_BR1$, $GABA_BR2$, and corresponding α-Tubulin in WT and *Fmr1* KO mice are shown for Figure 5. (c1-3) Blot was probed for (c1) rabbit anti-$GABA_BR1$ and (c2) mouse anti-$GABA_BR2$ simultaneously and was cut just below 75kD and probed for (c3) α-Tubulin. Molecular weight marker is shown to the left of all images. (d1-2) Full representative gels showing RT-qPCR amplified product of input sample, FMRP RIP, and IgG control are shown for (d1) $GABA_BR1$ and (d2) $GABA_BR2$.
